# Numerical investigation on performance of concrete-steel composite beams incorporating multi-transverse holes

**DOI:** 10.1038/s41598-025-32044-4

**Published:** 2026-01-06

**Authors:** Sabry Fayed, Mohamed Ghalla, Ehab A. Mlybari, Rabeea W. Bazuhair, Abdulaziz Alaskar, Saad A. Yehia

**Affiliations:** 1 Department of Civil Engineering, Faculty of Engineering, Kafrelsheikh University, Kafr El-Shaikh, Egypt; 2 Department of Civil Engineering, College of Engineering and Architecture, Umm Al-Qura University, Mecca, Saudi Arabia; 3 Department of Civil Engineering, College of Engineering, King Saud University, 11421 Riyadh, Saudi Arabia; 4 Civil Engineering Department, Higher Institute of Engineering and Technology, Kafr El-Shaikh, Egypt

**Keywords:** FEA, Composite beams, Shear, Multi-transverse holes, I-shaped sections, Ultimate load, Deflection, Engineering, Materials science

## Abstract

The integration of web openings in reinforced concrete (RC) beams for building services severely compromises shear capacity by disrupting load paths and creating critical stress concentrations. While previous research has focused on external strengthening of traditional RC beams, a significant gap exists regarding the performance of composite beams with embedded steel sections near openings. This study introduces a novel strengthening strategy using internally built-up I-section and T-section steel elements as shear reinforcement. The methodology integrated experimental testing with a validated nonlinear 3D finite element model in ABAQUS to conduct an extensive parametric study. Key investigated parameters included I-section web thickness (0.1–2.5 mm) and flange width (16–64 mm), and T-section compression and tension flange widths (0–64 mm). The key findings were substantial: web openings caused a 14% reduction in strength but a 41% increase in ductility, indicating a brittle failure mode. The incorporation of steel sections effectively reversed this; the I-section (176 × 2 mm web, 40 × 2 mm flanges) in beam BI-W2.0 yielded a remarkable 53.4% increase in ultimate load capacity, outperforming the original solid beam. An optimal I-section web thickness of 2.0 mm was identified, with diminishing returns beyond this point. For T-sections, the tension flange width was far more influential than the compression flange on strength recovery. A fundamental finding was that even a simple steel web alone provided a 43% strength gain, highlighting the critical role of bridging the opening. The reinforcement trade-off was a controlled 16–22% reduction in deflection, enhancing stiffness while maintaining structural safety. The research provides optimized, practical design guidelines for utilizing built-up steel sections to ensure structural integrity in perforated beams, effectively bridging architectural functionality and engineering safety.

## Introduction

The integration of web openings in reinforced concrete (RC) beams has emerged as a fundamental design strategy in modern construction, particularly in facilities such as hospitals, administrative buildings, and commercial centers. These openings facilitate the passage of essential mechanical, electrical, and communication systems, including high- and low-voltage cabling, data networks, HVAC ducts, and fire protection installations^1–4^. By eliminating the need to lower ceiling levels, web openings contribute to reduced overall building height, improved spatial efficiency, cost optimization, and the enhancement of interior architectural quality^5^. Although web openings are essential for modern service integration, their presence alters the structural behavior of beams, notably reducing shear strength and increasing the risk of localized failure. The introduction of web openings in concrete beams disrupts the shear transfer mechanism, significantly reducing the member’s capacity in the affected region. These discontinuities generate high stress concentrations around the opening, potentially leading to structural issues such as cracking or failure. Effective design practices are essential to preserve the shear strength of such elements^6^.

Given the detrimental impact of web openings on the shear capacity and overall structural performance of RC beams, it is essential to adopt effective mitigation strategies. Addressing these structural vulnerabilities requires not only a thorough understanding of the stress redistributions caused by the openings but also the implementation of targeted strengthening techniques. In particular, reinforcement becomes critical in high-stress regions or near supports, where the risk of localized failure is elevated. Common approaches to restoring shear capacity include the use of steel plates, perimeter stiffeners, and geometric modifications to reduce stress concentrations. Additionally, incorporating high-strength materials can further enhance local resistance^7–10^. The success of these interventions depends on accurate identification of load paths and effective force redistribution. Therefore, strict adherence to design standards, supported by advanced analytical and numerical modeling methods, is imperative to ensure structural safety while accommodating architectural and functional demands^11^. In this context, extensive experimental and analytical investigations have been carried out to elucidate the structural behavior of RC beams with web openings under diverse loading conditions. In an experimental study, Elansary et al.^12^ analyzed the impact of web openings near beam supports on shear strength. Testing six RC beams of different opening sizes revealed that larger openings caused up to a 35% reduction in shear capacity. However, CFRP sheet reinforcement restored performance, enhancing shear strength by 21–28%. In^7^, Mansour utilized an ABAQUS-based finite element model, validated against experimental data, to examine the shear strengthening effects of FRP layers on continuous RC beams with web openings. The research focused on the influence of opening location, area, and strengthening schemes. The most pronounced load reduction (66.1%) was due to opening placement, while comprehensive FRP coverage yielded the highest load capacity.

In another study, Özkılıç et al.^9^ used ABAQUS to analyze RC beams with circular openings strengthened by CFRP, focusing on diameter-to-height ratio, concrete strength, stirrup spacing, and CFRP methods. They found CFRP layering and application minimally affected beams with D/H ≤ 0.44, but full wrapping was necessary for D/H > 0.64. Concrete strength and stirrup spacing influenced performance, with four CFRP layers optimal for D/H above 0.44. Hakeem et al.^13^ examined the efficacy of steel plates and steel boxes in restoring shear capacity of composite beams with large web openings. Six HSSCC beams with different steel plate lengths were tested, with 680 mm plates providing a 116% increase in ultimate load. A validated ABAQUS finite element model was employed for parametric studies. Elsayed et al.^14^ performed experimental tests on UHPC beams featuring web openings, varying fiber content, reinforcement, and opening characteristics. Web openings decreased shear strength by 33.7%, but fiber reinforcement and additional strengthening significantly improved performance. Their analytical model and empirical equation accurately reflected experimental data.

Furthermore, to enhance RC beam performance under diverse loading types—including shear, bending, torsion, and cyclic loads—various reinforcement strategies have been developed. These include the use of steel bars, mesh fabrics, and steel plate systems. Stolarski and Zychowicz^15^ demonstrated that a truss-shaped reinforcement model significantly increased strength and minimized cracking relative to standard configurations. Shaheen and Mahmoud^16^ observed that expanded steel mesh outperformed welded mesh by 16%, while fiberglass mesh reduced capacity by 38%. Suwanpanjasil et al.^17^ applied PBO fiber mesh as shear reinforcement, finding increased capacity with more mesh layers and tighter spacing. Abd et al.^18^ utilized textile carbon yarns and noted significant gains when combined with steel fibers and optimally aligned. Al-Rousan^19^ emphasized the effectiveness of welded wire mesh in maintaining shear strength at elevated temperatures. Li et al.^20^ introduced CFRP-MF as an alternative to steel stirrups, confirming its code-compliant performance through FEM simulations. In addition, Fayed et al.^21^ evaluated the impact of steel plates around web openings in RC beams. Although increased thickness reduced effectiveness, longitudinal SP arrangements improved peak load by 69%. A finite element study further indicated that increasing the SP-to-depth ratio yielded a 141% gain in shear capacity.

While significant advancements have been made in understanding the behavior of RC beams with web openings and in developing effective strengthening strategies—such as CFRP wrapping, fiber meshes, and steel plate reinforcement—most existing studies focus on traditional RC beams. Limited research has been conducted on composite beams composed of concrete and embedded steel elements, particularly in the presence of web openings. These composite configurations present more complex mechanical behavior due to the interaction between steel and concrete under shear loads, especially when openings are introduced. Moreover, although experimental and numerical studies have assessed the influence of opening size, location, and reinforcement type on conventional RC beams, the performance of hybrid composite beams with embedded metallic reinforcement around openings remains largely unexplored. Existing design codes also offer insufficient guidance for predicting the structural response of such systems, particularly in shear-critical regions. This lack of comprehensive data and analytical modeling for composite beams with web openings under shear loading conditions presents a critical research gap. Addressing this gap is essential for developing reliable design strategies and improving the structural safety and efficiency of modern constructions that incorporate integrated service systems. Accordingly, the current study comprises experimental testing of three reinforced concrete beam specimens, complemented by a validated numerical model, to evaluate the structural behavior of beams with multiple web openings strengthened using built-up steel elements in I- and T-section forms as alternatives to conventional shear reinforcement.

## Experimental work

In this work, one reference solid beam and two concrete beams with several transverse holes were cast and subjected to flexure loading tests. Portland cement (grade 42.5 MPa), water, crushed dolomite (with a maximum size 12 mm) as rough aggregate, and river sandy as fine aggregate were all included in the concrete mix used in this project. These combined materials include 700 kg of dolomite per cubic meter, 660 kg of sand per cubic meter, 350 kg of cement per cubic meter, and 175 L of water per cubic meter. After 28 days, three standard cubes had been examined to ascertain their compressive strength. 25.8 MPa was the average highest strength (Table 1). As a tension longitudinal flexural reinforcing, 16 mm diameter high tensile steel rebars were employed. Tensile examinations were performed to ascertain their mechanical characteristics. With a modulus of elasticity of 205 GPa, a maximum tensile force of 550 MPa, and a mean yield value of 431 MPa, the tested bars demonstrated properties typical of high-strength steel (Table 1).

**Table 1:** Properties of materials used.

Material	Description	Compressive strength	Modulus of elasticity	Tensile strength	Yield stress
Concrete	Mix composed of cement, water, dolomite and sand	25.8 MPa	N/A	N/A	N/A
Flexure bars 16 mm	High tensile steel rebars having deformed surface	N/A	205 GPa	550 MPa	431 MPa
I-shaped steel section	I-shaped steel section	N/A	200 GPa	370 MPa	240 MPa
Steel mesh	4 mm-diameter steel wires that intersected in two orthogonal directions	N/A	200 GPa	388 MPa	255 MPa

**Fig. 1:**
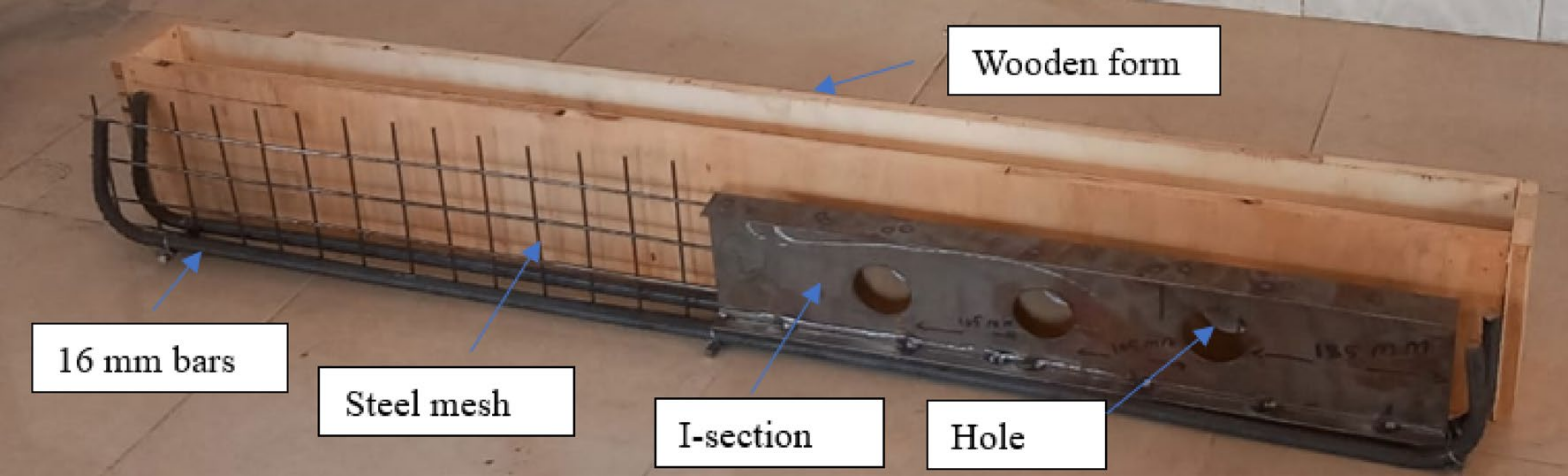
Materials used in beam BI-W2.0

The beam span was split into two equal shear spans, each measuring 700 mm, for shear areas. Steel mesh was used to bolster the left distance. The 4 mm-diameter steel wires that intersected to form the steel mesh were spaced 30 mm in the x-direction and 40 mm in the y-direction (Fig. 1). At every junction, this steel meshes were expertly welded. Three wires’ tensile tests verified the usual behavior of mild steel, with an elastic modulus of 200 GPa, an ultimate tensile strength of 388 MPa, and an average yield strength of 255 MPa (Table 1).

**Fig. 2:**
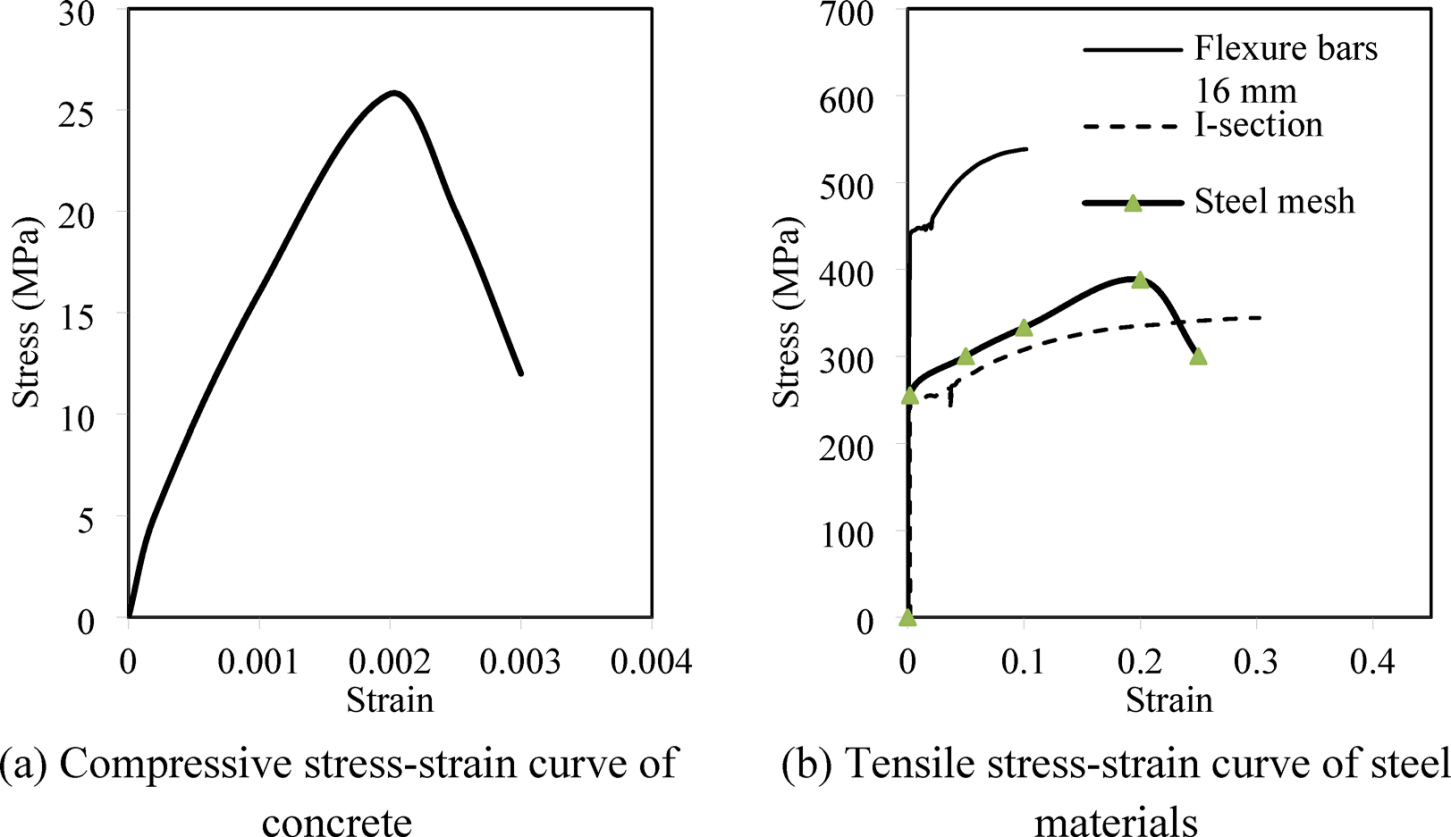
Stress–strain curve of materials used in the current study.

In the right shear spans of the tested beams, where the apertures are placed, an I-shaped steel section was used as reinforcement for shear (Fig. 1). According to tension testing, the I-section has a tension resistance of 370 MPa and a yield value of 240 MPa (Table 1). The web is 176 mm in height and is 2 mm thick, while the flange is 40 mm wide and 2 mm thick. Figure 2 shows true stress–strain curve of materials used in the current study.

**Fig. 3:**
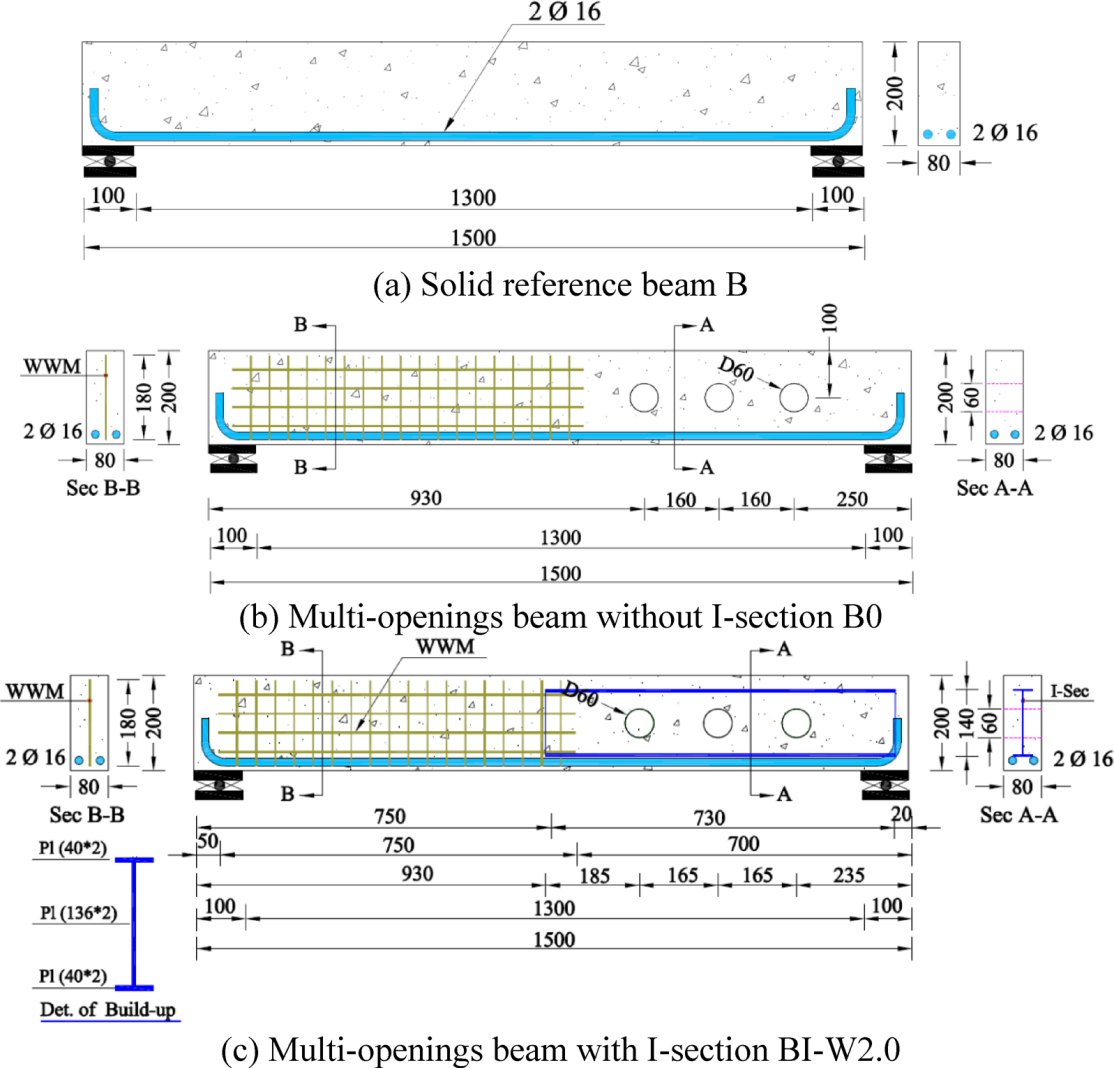
Details of tested beams.

Three reinforced concrete beams in all, each measuring 1500 mm in length and having the same 80 × 200 mm cross-section, were cast. The chosen shear span in this investigation was 700 mm, while the beam depth was 182 mm. Two 16 mm diameter tension bars were used to brace every beam in order to prevent flexural failure. Additionally, the beams in the right side were made to fail under shear. To guarantee that there would be no shear failure in this area, the left side of each beam—which was solid and devoid of openings—was heavily strengthened in shear using steel mesh. Only the right side of the beams, which had an I-shaped steel beam for reinforcement, had any web apertures. That was carried out in order to evaluate the performance of various I-shaped steel beam designs and investigate the behavior of shear failure. First tested and utilized as a reference model for comparison was the control beam (B), which is seen in Fig. 3a and has no holes or shear reinforcement. Three round apertures were present at the right shear span of beams B0 and BI-W2.0. Every hole had a round form and a fixed 60 mm diameter. In order to improve shear resistance, Beam BI-W2.0 was reinforced with an I-shaped section along half of its span, as seen in Fig. 3c. In contrast, Beam B0 had no I-section, as seen in Fig. 3b.

**Fig. 4:**
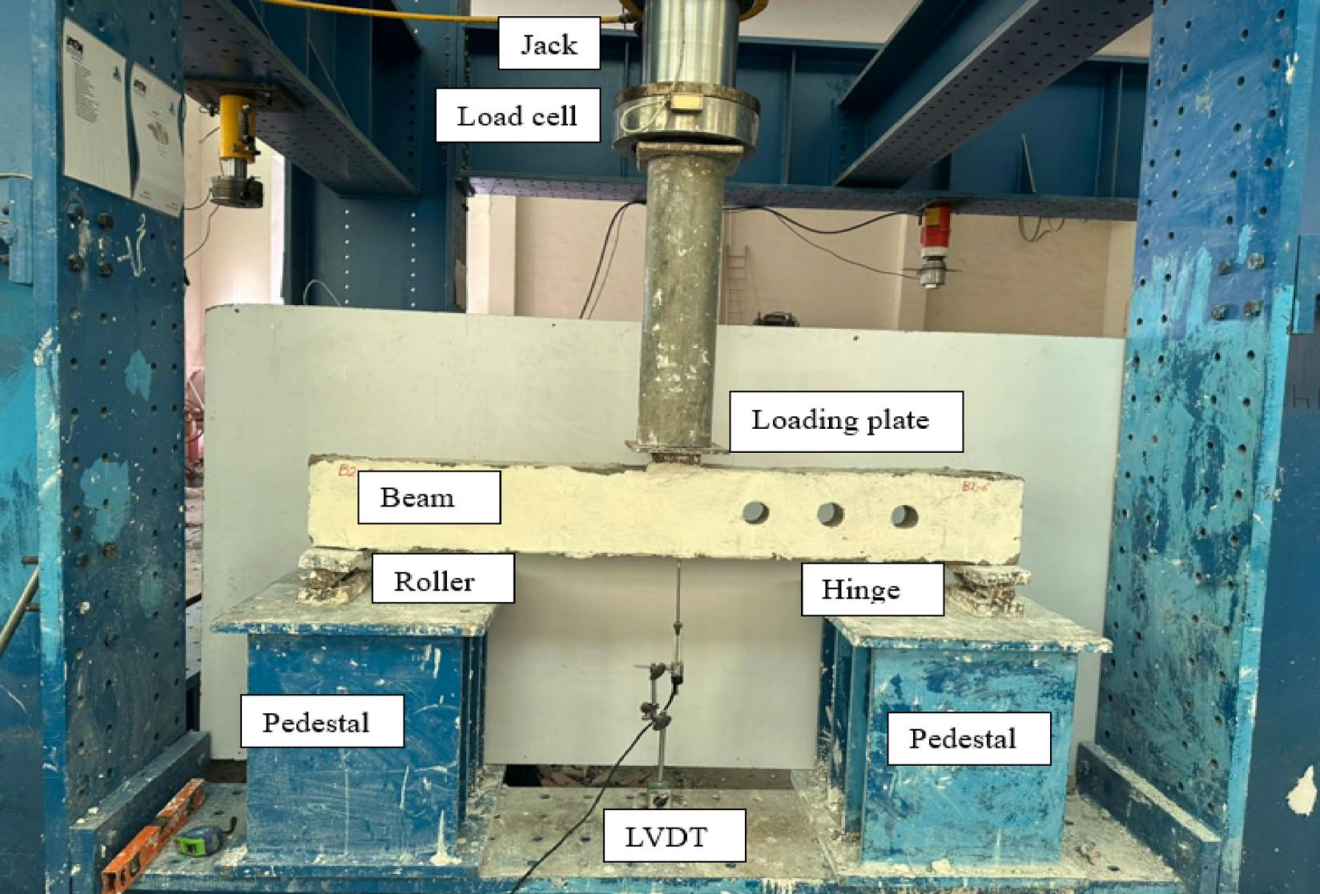
Test set-up.

As seen in Fig. 4, a universal hydraulic loading frame was used to conduct the beam testing. In the middle of the beams’ span, a vertical focused load was delivered via a hydraulic actuator. Steel blocks that allowed for free rotation served as supports, and an LVDT was positioned at the mid-span of the beam to detect vertical deflection.

## Numerical analysis

To investigate the shear response of composite RC beams with multiple web openings, a nonlinear three-dimensional finite element analysis was conducted using ABAQUS v.2021. This software was selected due to its robust capabilities in capturing complex loading paths, contact interactions, and nonlinear material behavior. The accuracy of the numerical model was validated against experimental results, demonstrating its reliability in predicting structural performance.

### FE model configuration

**Fig. 5:**
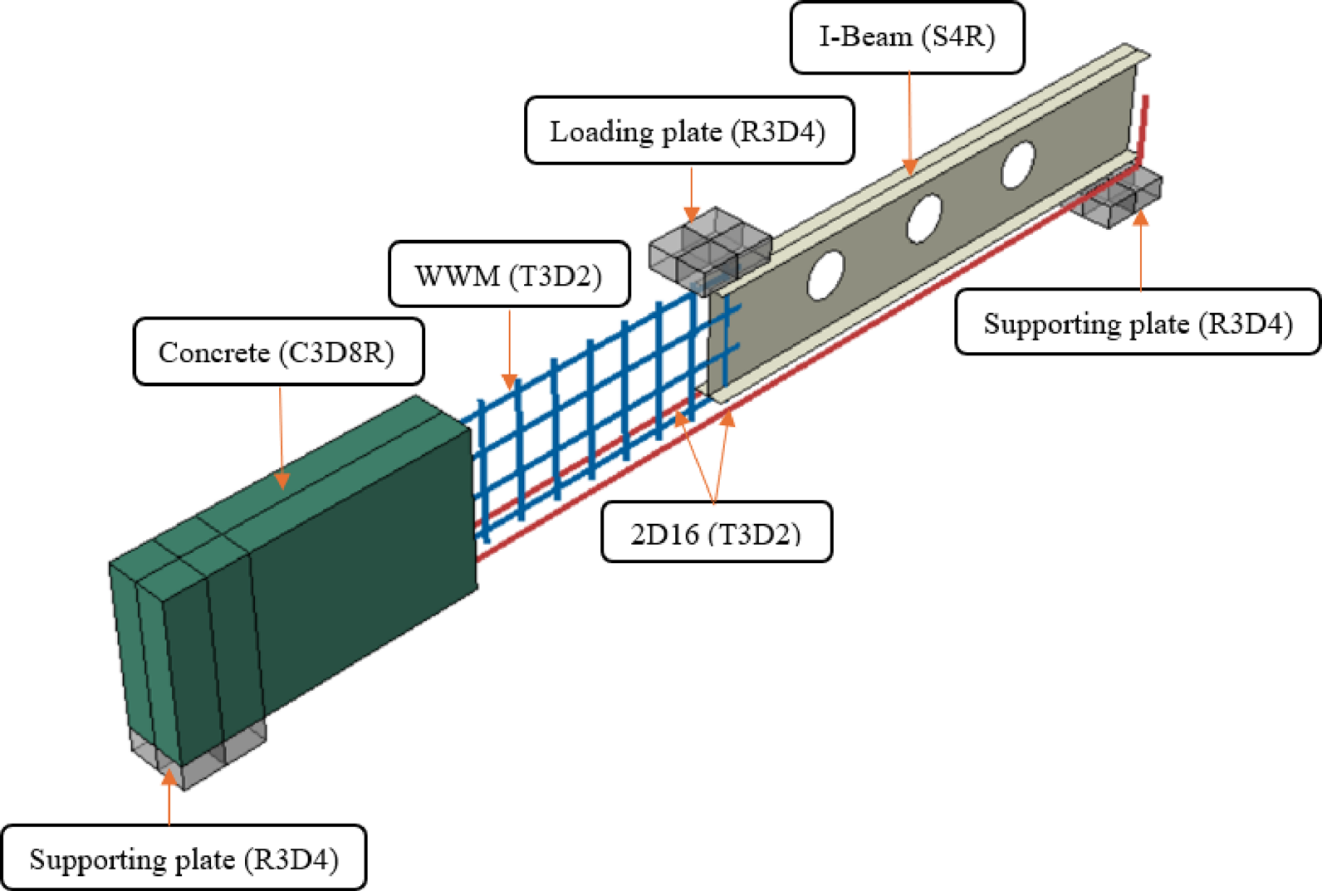
Element types of beam BI-W2.0

To replicate the structural behavior observed experimentally, a finite element model of beam BI-W2.0 was constructed (Fig. 5). Concrete was meshed with 8-node solid elements (C3D8R), while R3D4 rigid elements represented loading and support plates. Steel reinforcement and mesh were simulated via T3D2 elements, and the I-beam was modeled with S4R shell elements for efficient computation. Boundary conditions imitated experimental supports through central line constraints. Additionally, regarding initial geometric imperfections, small geometric deviations in the steel I-sections were naturally present due to fabrication tolerances. However, explicit initial imperfections were not incorporated into the numerical model.

**Fig. 6:**
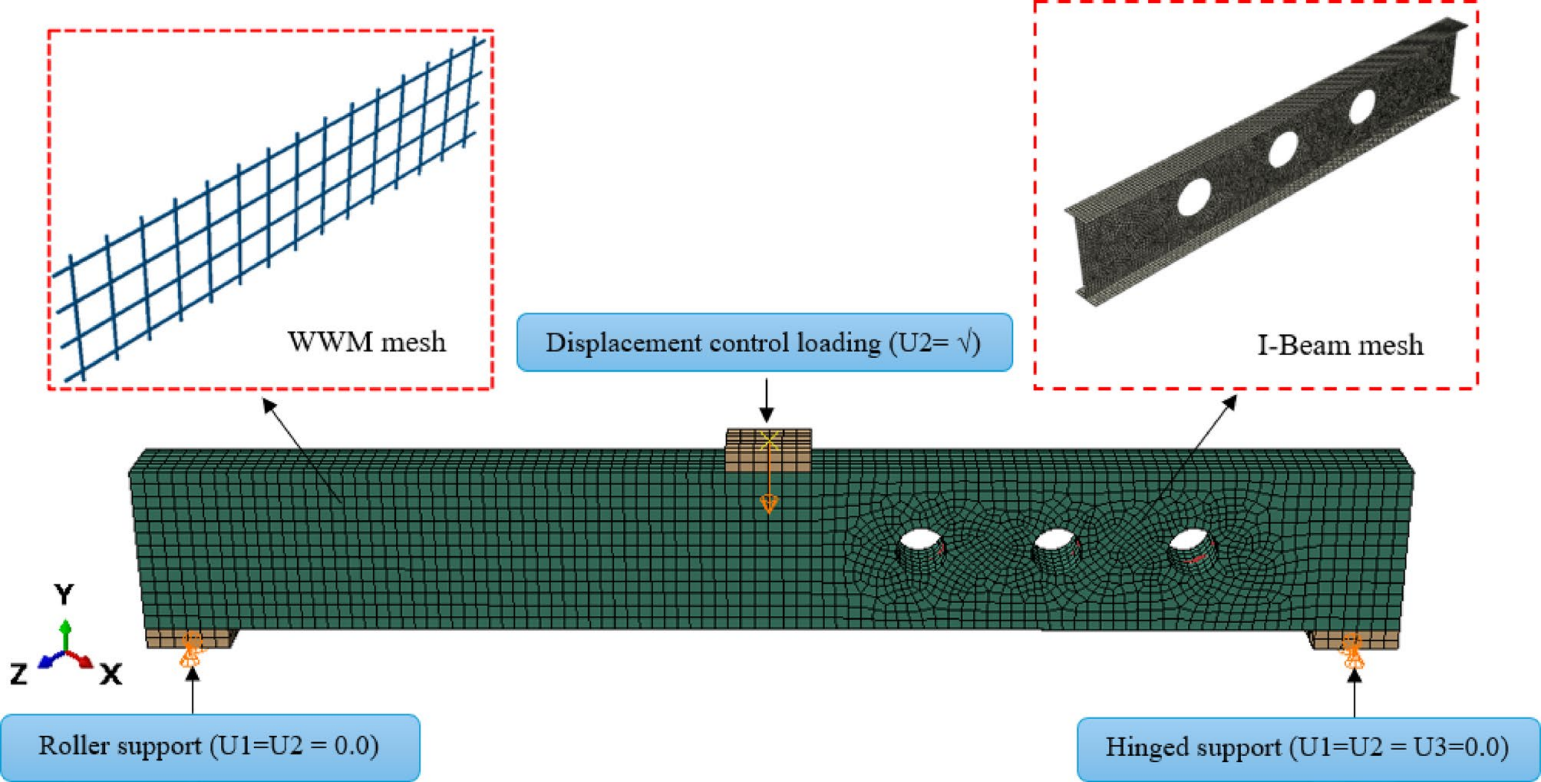
Typical meshing, boundary conditions, and loading of the of the tested beams.

The embedded region interaction technique was employed to represent the bond mechanism between the reinforcing steel and the surrounding normal concrete (NC). In this approach, the steel reinforcement is embedded directly within the host concrete mesh, ensuring that the translational degrees of freedom of the steel nodes are constrained to those of the concrete elements. This method effectively captures the full composite action between the two materials and prevents relative slip at the interface, providing a stable and computationally efficient representation of bond behavior within the finite element model. Loading was applied through two reference nodes at the plate midspan, coupled using multi-point constraints to achieve uniform distribution (Fig. 6).

**Fig. 7:**
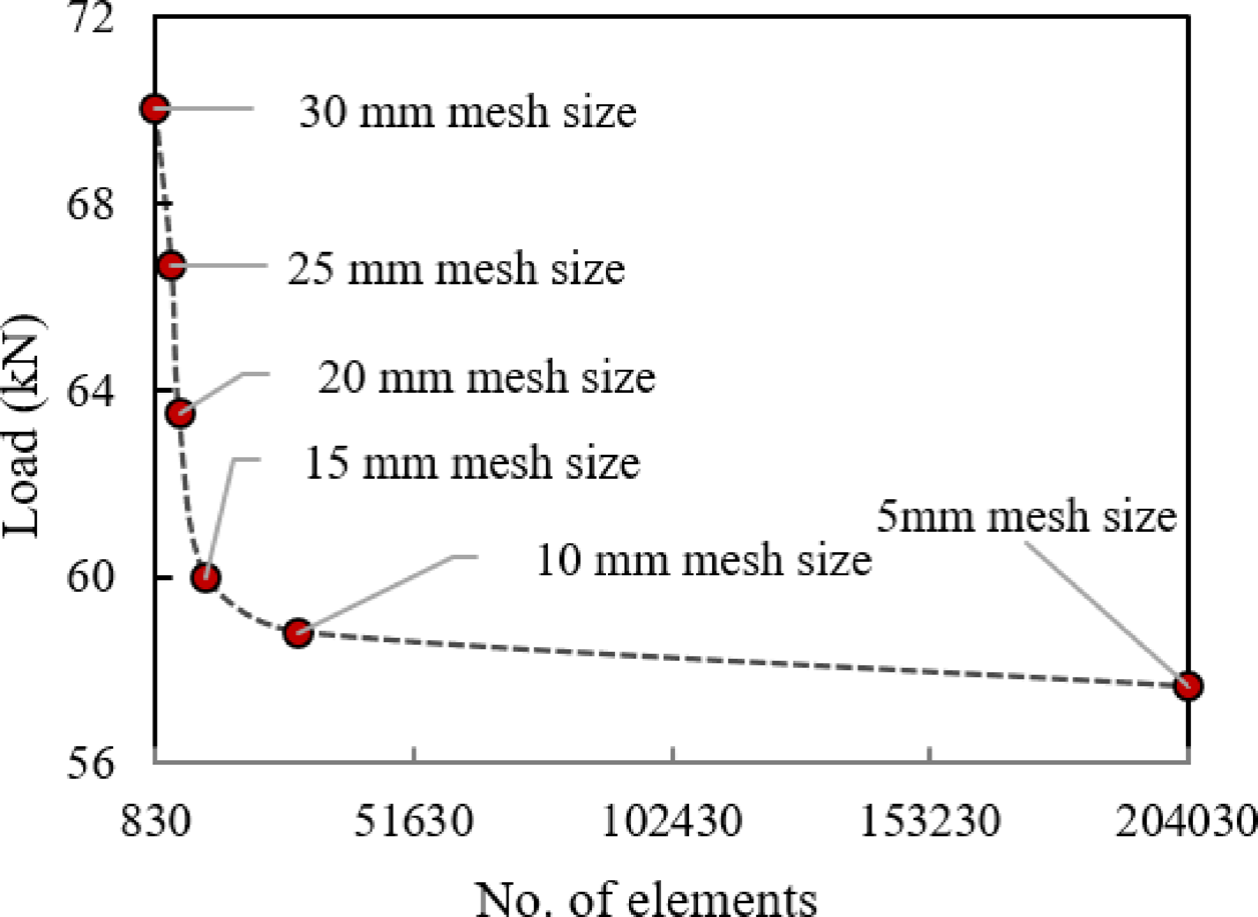
Load level for different mesh sizes.

A mesh sensitivity analysis was conducted to enhance the fidelity of the finite element model, with element sizes ranging from 5 to 30 mm evaluated against the corresponding experimental results (Fig. 7). The difference in predicted ultimate load between the 5 mm and 10 mm meshes was less than 2%, confirming that the 10 mm mesh provides an optimal balance between accuracy and computational efficiency for both the concrete and steel reinforcement. The finalized BI-W2.0 beam model consisted of 28,901 elements and 33,869 nodes.

### Modeling of material properties

#### Concrete

To capture the true mechanical behavior of normal concrete (NC), the Concrete Damage Plasticity (CDP) model was adopted, which requires compressive and tensile behavior input parameters^22–36^. Under tensile loading, the stress–strain relationship remains linear up to the failure stress, after which softening occurs due to micro-cracking. In compression, the material response is linear up to the yield stress, followed by a hardening phase, then softening beyond the ultimate compressive strength. ABAQUS facilitates realistic simulation by converting user-defined uniaxial stress–inelastic strain data into stress–plastic strain curves, ensuring reliable representation of the complex damage mechanics in concrete structures, as shown in Eqs. (1) and (2).

1 $$\sigma_{t} = \sigma_{t} \left( {\varepsilon_{t}^{pl} , \varepsilon_{t}^{^{\prime} pl} } \right)$$

2 $$\sigma_{c} = \sigma_{c} \left( {\varepsilon_{c}^{pl} , \varepsilon_{c}^{^{\prime} pl} } \right)$$

Tension and compression are indicated by subscripts *t* and *c*. The concrete stresses under these conditions are defined as by $\sigma_{t}$ and $\sigma_{c}$. $\varepsilon_{t}^{pl}$ and $\varepsilon_{c}^{pl}$ represent the equivalent plastic strains, while $\varepsilon_{t}^{^{\prime}pl}$ and $\varepsilon_{c}^{^{\prime}pl}$ refer to their respective strain rates. The damage formulations for tension and compression proposed by Birtel and Mark^37^ were adopted to simulate the concrete failure mechanisms under respective loading conditions, as described in Eqs. (3) and (4).

3 $$d_{t} = 1 - \frac{{\sigma_{t} E_{c}^{ - 1} }}{{\varepsilon_{t}^{pl} (1/b_{t} - 1) + \sigma_{t} E_{c}^{ - 1} }}$$

4 $$d_{c} = 1 - \frac{{\sigma_{c} E_{c}^{ - 1} }}{{\varepsilon_{c}^{pl} (1/b_{c} - 1) + \sigma_{c} E_{c}^{ - 1} }}$$

where the tensile and compressive damage parameters are denoted by $d_{t}$ and $d_{c}$, respectively. The constant parameters, $b_{t}$ and $b_{c}$, range between 0 and 1.

The Saenz model^38^ was utilized to provide a detailed representation of concrete behavior under uniaxial compression, as outlined in Eqs. (5)–(11).

5 $$\sigma_{c} = \frac{{E_{c} \varepsilon_{c} }}{{1 + \left( {R + R_{E} - 2} \right)\frac{{\varepsilon_{c} }}{{\varepsilon_{o} }} - \left( {2R - 1} \right)\left( {\frac{{\varepsilon_{c} }}{{\varepsilon_{o} }}} \right)^{2} + R\left( {\frac{{\varepsilon_{c} }}{{\varepsilon_{o} }}} \right)^{3} }}$$

6 $$E_{c} = 4700\sqrt {f_{c}{\prime} }$$

for normal strength concrete^39^

7 $$R = \frac{{R_{E} \left( {R_{\sigma } - 1} \right)}}{{\left( {R_{\varepsilon } - 1} \right)^{2} }} - \frac{1}{{R_{\varepsilon } }}$$

8 $$R_{E} = \frac{{E_{C} }}{{E_{O} }}$$

9 $$R_{\sigma } = \frac{{f_{c}{\prime} }}{{\sigma_{f} }}$$

10 $$R_{\varepsilon } = \frac{{\varepsilon_{f} }}{{\varepsilon_{o} }}$$

11 $$E_{O} = \frac{{f_{c}{\prime} }}{{\varepsilon_{O} }}$$

where $\sigma_{c}$ was the compression strength of the concrete, $E_{c}$ was concrete elasticity index, $E_{O}$ was concrete secant elasticity, $f_{c}{\prime}$ was ultimate compression, $\varepsilon_{c}$ was the strain value in the compression; $\varepsilon_{o}$ was corresponding strain at the $f_{c}{\prime}$ value (equals about 0.0025^40^), $\varepsilon_{f}$ was peak strain, $\sigma_{f}$ was pressure related to $\sigma_{f}$, $R_{E}$ was modular factor, $R_{\sigma }$ was ratio of the pressure (taken 4^40^), $R_{\varepsilon }$ was ratio of strain (taken 4^40^).

The uniaxial tensile stress–strain behavior of concrete was characterized by two phases: an initial linear elastic response and a subsequent softening curve. For an initial linear elastic response, the elastic modulus ($E_{C}$) and tensile strength ($\sigma_{to}$) were critical for characterizing the linear response of each material. Specifically, for concrete, the elastic modulus $E_{C}$ was defined according to Eq. 6. while the tensile softening branch was calculated using Eq. 12, following the methodology outlined by Ganganagoudar et al.^41^.

12 $$\sigma_{t} = \sigma_{to} e^{{\left[ {350\left( {\frac{{\sigma_{to} }}{{E_{c} }} - \varepsilon_{t} } \right)} \right]}}$$

where $\varepsilon_{t}$ denotes the tensile strain associated with $\sigma_{t}$, the tensile strength.

The Concrete Damaged Plasticity (CDP) framework relies on four fundamental input parameters: dilation angle, eccentricity (ε), the yield surface shape factor $K_{c}$, and the strength ratio $f_{bo} /f_{co}$​. The dilation angle defines the slope of the failure surface in the meridional plane, whereas ε controls the rate of approach to this asymptote. The $K_{c}$ parameter adjusts the shape of the deviatoric yield surface, and the strength ratio indicates the relative compressive capacity under biaxial and uniaxial loading. For this study, the parameters were set as 26, 0.1, 0.67, and 1.16, respectively^42–45^. A viscosity parameter of 0.0001 was also introduced to address potential convergence problems and to alleviate mesh dependency issues.

#### Reinforcement material

To simulate reinforcement behavior under loading, a bilinear elastic–plastic model incorporating isotropic hardening was employed. The stress–strain relationship followed Han and Huo’s two-segment approach^46^. In the elastic range, stiffness and lateral contraction were defined by experimentally determined elastic modulus and Poisson’s ratio. Beyond yielding, a hardening modulus equal to 1% of the elastic modulus was introduced to capture strain hardening. Isotropic hardening facilitated accurate simulation of post-yield behavior by enabling uniform expansion of the yield surface. This method effectively captured the nonlinear response under both monotonic and cyclic loading. Material properties were mathematically formulated following the model presented in^47^.

13 $$\sigma_{i} = E_{s} \varepsilon_{s} \quad for\;\varepsilon_{s} \le \varepsilon_{sy} \quad \left( {elastic\;region} \right)$$

14 $$\sigma_{i} = f_{sy} + E_{p} \left( {\varepsilon_{s} - \varepsilon_{sy} } \right)\quad for\;\varepsilon_{s} \le \varepsilon_{sy} \quad \left( {plastic\;region} \right)$$

In ABAQUS, steel was modeled as linearly elastic until the yield point and plastic beyond it. The required parameters—$\sigma_{i}$, $E_{s}$, $\varepsilon_{s}$, $f_{sy}$, and $\varepsilon_{sy}$—were used to define the material law. Engineering stress–strain data were converted to true values using equations from^48^:

15 $$f_{tr} = f_{norm} \left( {1 + \varepsilon_{norm} } \right)$$

16 $$\varepsilon_{tr}^{pl} = ln\left( {1 + \varepsilon_{norm} } \right) - f_{tr} /E_{s}$$

In this context, $f_{tr}$ refers to the true stress and $f_{norm}$ to the nominal (engineering) stress. Similarly, $\varepsilon_{tr}^{pl}$ indicates the true plastic strain, and $\varepsilon_{norm}$ represents the nominal strain.

### FEA verification of P-δ curve and failure modes

**Fig. 8:**
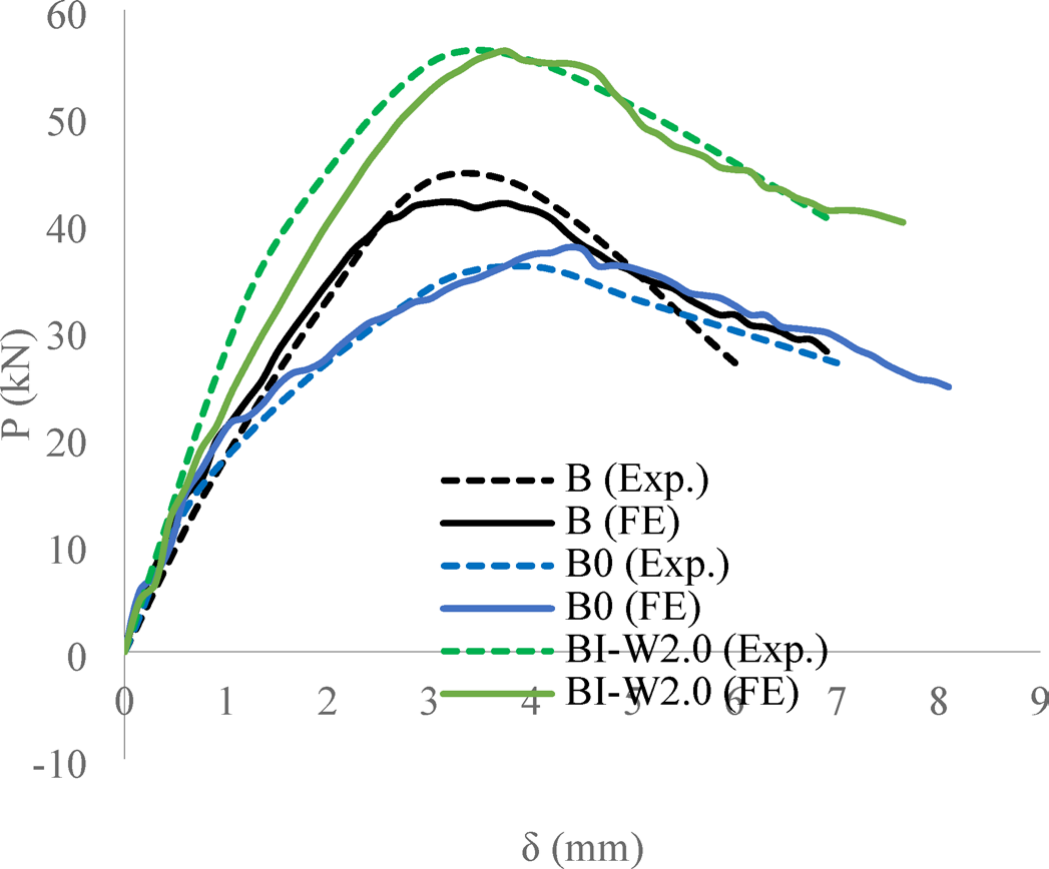
Load–deflection curve of three beams, obtained by experiments and FEA (ABAQUS).

**Fig. 9:**
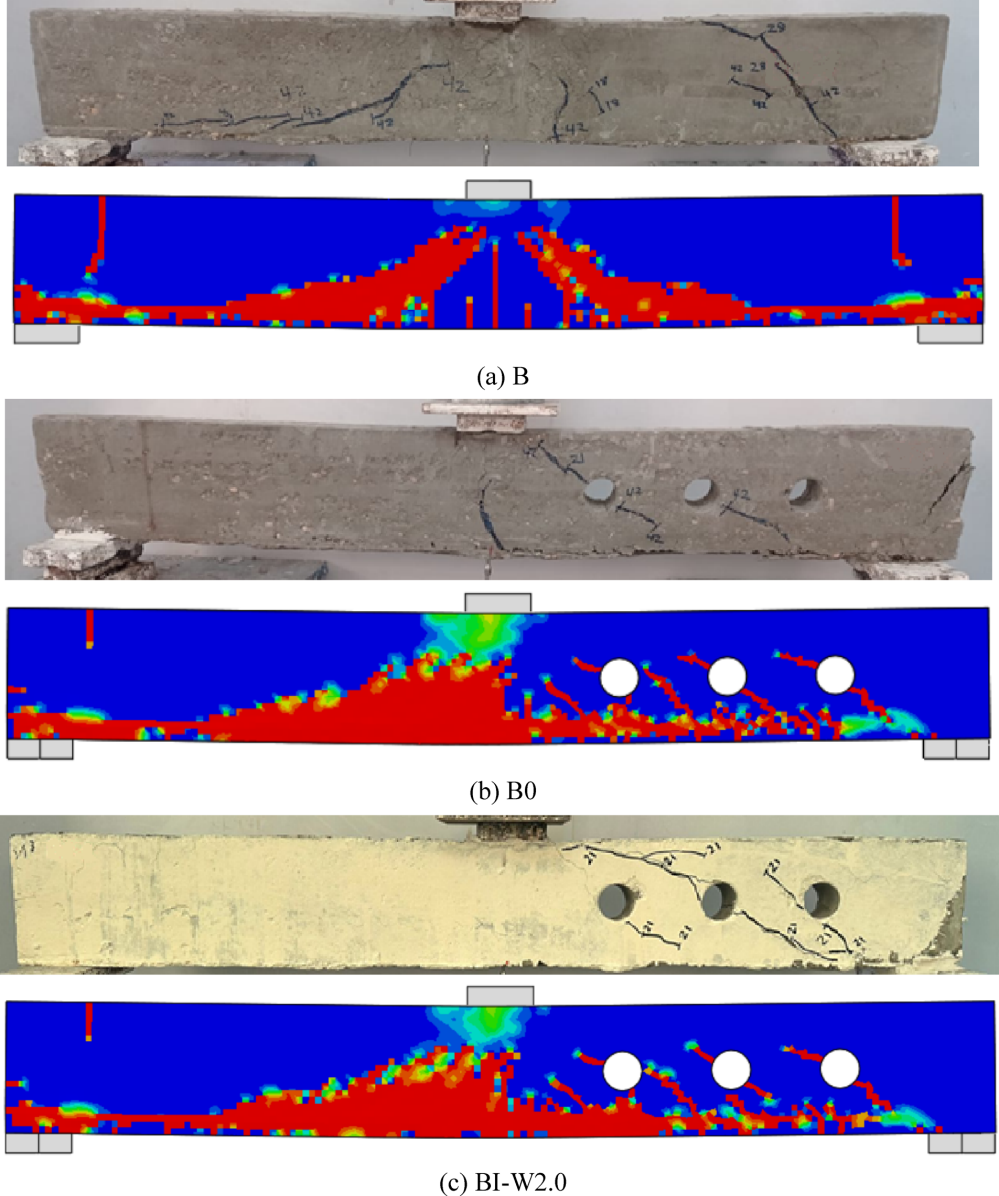
Failure modes of three beams, obtained by experiments and FEA (ABAQUS).

The numerical simulations showed excellent agreement with the experimental results in terms of overall load–deflection behavior and crack development (Figs. 8 and 9). As summarized in Table 2, the FEM successfully reproduced all observed failure mechanisms and accurately captured the effects of different reinforcement configurations. Beam B-I-W2.0 exhibited a higher ultimate load than the solid reference beam B (56 kN versus 44 kN), representing an increase of 27%. This can be attributed to the fact that the web openings significantly reduce the shear capacity and stiffness of the beam, while the I-shaped section primarily compensates for this loss and provides additional capacity beyond that of the solid beam. Consequently, the strengthened beam restores the capacity lost due to the openings and slightly exceeds the inherent strength of the solid beam without voids. The similar deflection values align with this mechanism, as the concrete section and tension reinforcement dominate the overall flexural stiffness, while the I-section mainly enhances shear resistance near the openings. The very low experimental-to-simulation dispersion (0.953 for peak load and 0.967 for deflection, with CoV values of 0.002 and 0.057) further validates this explanation and confirms the reliability of the numerical model in capturing the observed behavior. Additionally, although explicit initial imperfections were not modeled, the FE model accurately reproduced the experimental behavior, indicating that these minor deviations did not influence the overall conclusions.

**Table 2:** Summary of experimental and numerical performance metrics.

Beam`s ID	${P}_{U}$			${\delta }_{u}$		
	$Exp$	$FE$	$Exp/FE$	$Exp$	$FE$	$Exp/FE$
B	44.0	46.05	0.955	3.0	3.15	0.952
B0	36.0	37.8	0.952	4.0	4.35	0.920
B-I-W2.0	56.0	58.8	0.952	3.7	3.60	1.028
µ			0.953			0.967
SD			0.002			0.055
CoV			0.002			0.057

µ: Standard average; SD: Coefficient of deviation; CoV: Coefficient of variation.

### Numerical parametric variables

To evaluate the structural behavior of RC beams with multiple web openings strengthened by built-up steel elements, a comprehensive numerical parametric study was conducted using validated finite element models. The test matrix, presented in Table 3, is organized into six groups (GI–GVI), each designed to investigate a specific variable affecting shear performance. Two types of built-up steel reinforcement sections were utilized in the study: I-sections (I-Sec) and T-sections (T-Sec), with varied geometric configurations aimed at optimizing structural behavior.

**Table 3:** Test matrix.

Group	Beam`s ID	Group aim	Build-up section configuration							
			I-Sec (Dim. mm)				T-Sec (Dim. mm)			
			Web		Flange		Web		Flange	
			Length	Thick	Width	Thick	Length	Thick	Width	Thick
GI	B	Effect of existing multi-openings	–	–	–	–	–	–	–	–
	B0		–	–	–	–	–	–	–	–
GII	B0	Effect existing of I -Sec	–	–	–	–	–	–	–	–
	BI-W2.0		176.0	2.0	40.0	2.0	–	–	–	–
GIII	BI-W0.1	Effect of web thickness of the I-Sec	176.0	**0.1**	40.0	2.0	–	–	–	–
	BI-W0.2		176.0	**0.2**	40.0	2.0	–	–	–	–
	BI-W0.5		176.0	**0.5**	40.0	2.0	–	–	–	–
	BI-W1.0		176.0	**1.0**	40.0	2.0	–	–	–	–
	BI-W1.5		176.0	**1.50**	40.0	2.0	–	–	–	–
	BI-W2.0		176.0	**2.0**	40.0	2.0	–	–	–	–
	BI-W2.5		176.0	**2.50**	40.0	2.0	–	–	–	–
GIV	BT-CF0	Effect of compression flange width of the T -Sec	–	–	–	–	176.0	2.0	**0.0**	0.0
	BT-CF0.2		–	–	–	–	176.0	2.0	**16.0**	2.0
	BT-CF0.4		–	–	–	–	176.0	2.0	**32.0**	2.0
	BT-CF0.6		–	–	–	–	176.0	2.0	**48.0**	2.0
	BT-CF0.8		–	–	–	–	176.0	2.0	**64.0**	2.0
GV	BT-TF0	Effect of tension flange width of the T -Sec	–	–	–	–	176.0	2.0	**0.0**	0.0
	BT-TF0.2		–	–	–	–	176.0	2.0	**16.0**	2.0
	BT-TF0.4		–	–	–	–	176.0	2.0	**32.0**	2.0
	BT-TF0.6		–	–	–	–	176.0	2.0	**48.0**	2.0
	BT-TF0.8		–	–	–	–	176.0	2.0	**64.0**	2.0
GVI	BI-F0.2	Effect of flange width of the I -Sec	176.0	2.0	**16.0**	2.0	–	–	–	–
	BI-F0.4		176.0	2.0	**32.0**	2.0	–	–	–	–
	BI-F0.6		176.0	2.0	**48.0**	2.0	–	–	–	–
	BI-F0.8		176.0	2.0	**64.0**	2.0	–	–	–	–

W: web; F: flange; CF: compression flange; TF: tension flange.

To improve clarity, all specimens were labeled according to the type of built-up section and the primary variable under investigation^49,50^.

- B denotes the baseline solid beam without openings.
- B0 represents the unstrengthened beam with multiple web openings.
- BI–W*X* refers to beams strengthened with an I-section, where *W* indicates the web thickness**,** and *X* is its numerical value in millimeters.
- BI–F*X* identifies beams strengthened with an I-section where *F* denotes flange width.
- BT–CF*X* and BT–TF*X* denote beams strengthened with T-sections, where *CF* and *TF* refer to compression and tension flange widths, respectively, and *X* indicates the flange width in millimeters.

This systematic notation provides a clear and consistent method for interpreting each specimen’s strengthening configuration. Group GI serves as the control series and includes two specimens: Beam B and Beam B0. These beams were unreinforced with any built-up steel sections. Beam B0 incorporated multiple web openings, representing a typical modern RC beam requiring service integration, whereas Beam B was constructed as a solid reference beam without any openings. This group provides the baseline against which the performance of reinforced configurations could be compared.

Group GII investigates the structural contribution of I-section steel elements as shear reinforcement for RC beams with web openings. The specimen labeled BI-W2.0 includes an I-section characterized by a 176 mm long web with a thickness of 2.0 mm, and flanges that are 40 mm wide and 2.0 mm thick. This configuration serves to establish the impact of adding steel reinforcement to beams that would otherwise suffer significant shear degradation due to web openings. Group GIII focuses on the effect of varying the web thickness in the I-section reinforcement. This group includes a series of specimens with web thicknesses ranging from 0.1 to 2.5 mm, while flange dimensions were held constant. The aim of this group is to determine the optimal web thickness that provides sufficient shear resistance while maintaining material efficiency. Group GIV explores the influence of the compression flange width in T-section reinforcement elements. In this group, the compression flange width of the T-section was incrementally increased from 0 to 64 mm. Throughout the group, the web dimensions and tension flange configuration were maintained constant. As the compression flange is located at the upper portion of the beam, the group evaluates how this geometric variation contributes to the redistribution of stresses and the enhancement of shear performance.

Group GV is designed to assess the effect of the tension flange width in T-section reinforcement elements. Similar to Group GIV, the tension flange width varied from 0 to 64 mm, while the web and compression flange dimensions were held constant. The tension flange is positioned at the bottom of the T-section, and this group seeks to determine its role in resisting shear failure and enhancing the ductility of the beam. Group GVI investigates the influence of flange width variations in I-section configurations. This series includes specimens with flange widths ranging from 16 to 64 mm, keeping the web thickness and length unchanged. By systematically varying flange dimensions, this group aims to determine the extent to which flange geometry contributes to shear capacity and overall beam performance.

## Results and discussion

### Failure modes

**Fig. 10:**
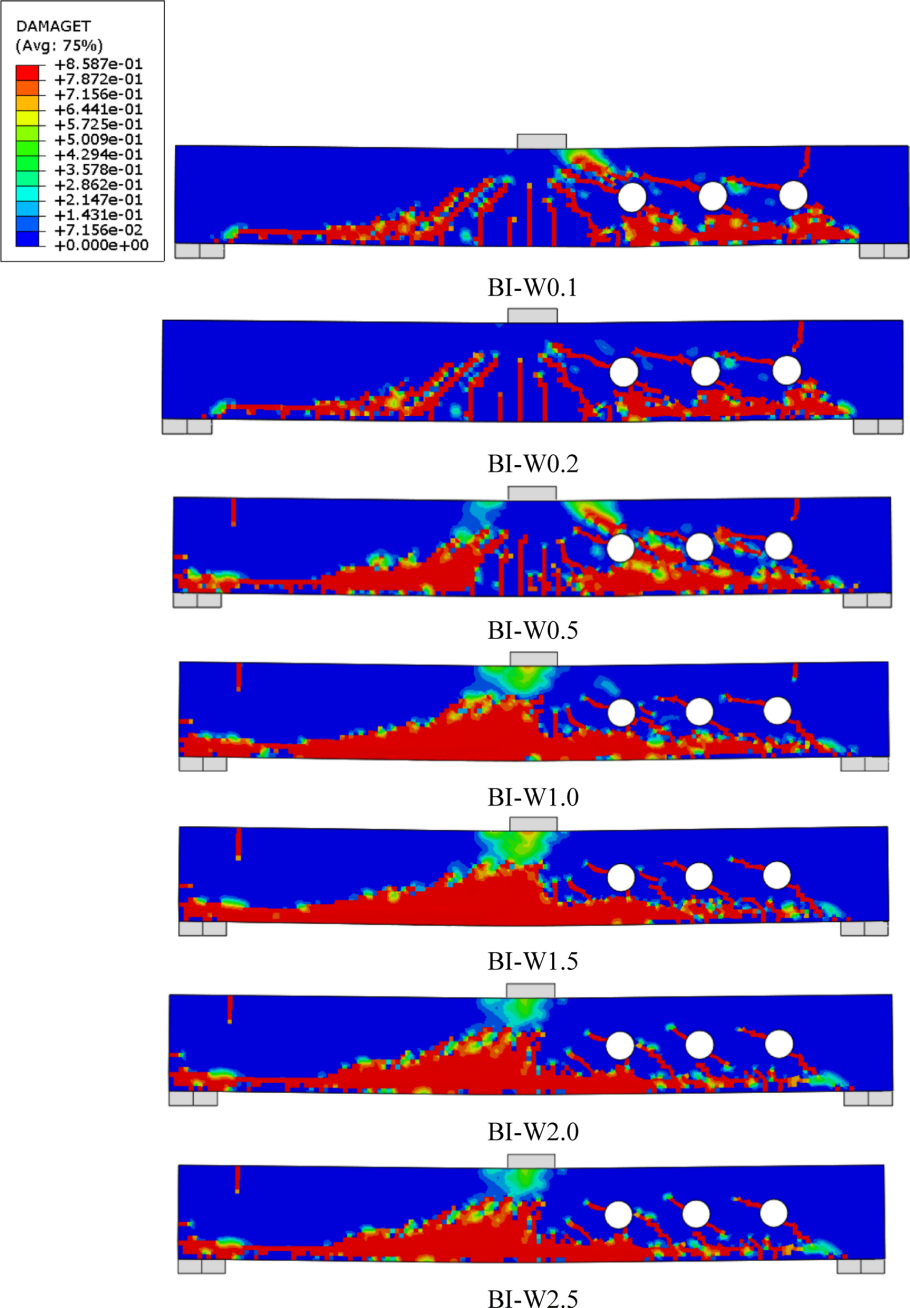
Failure patterns exhibited by Group GIII beams.

**Fig. 11:**
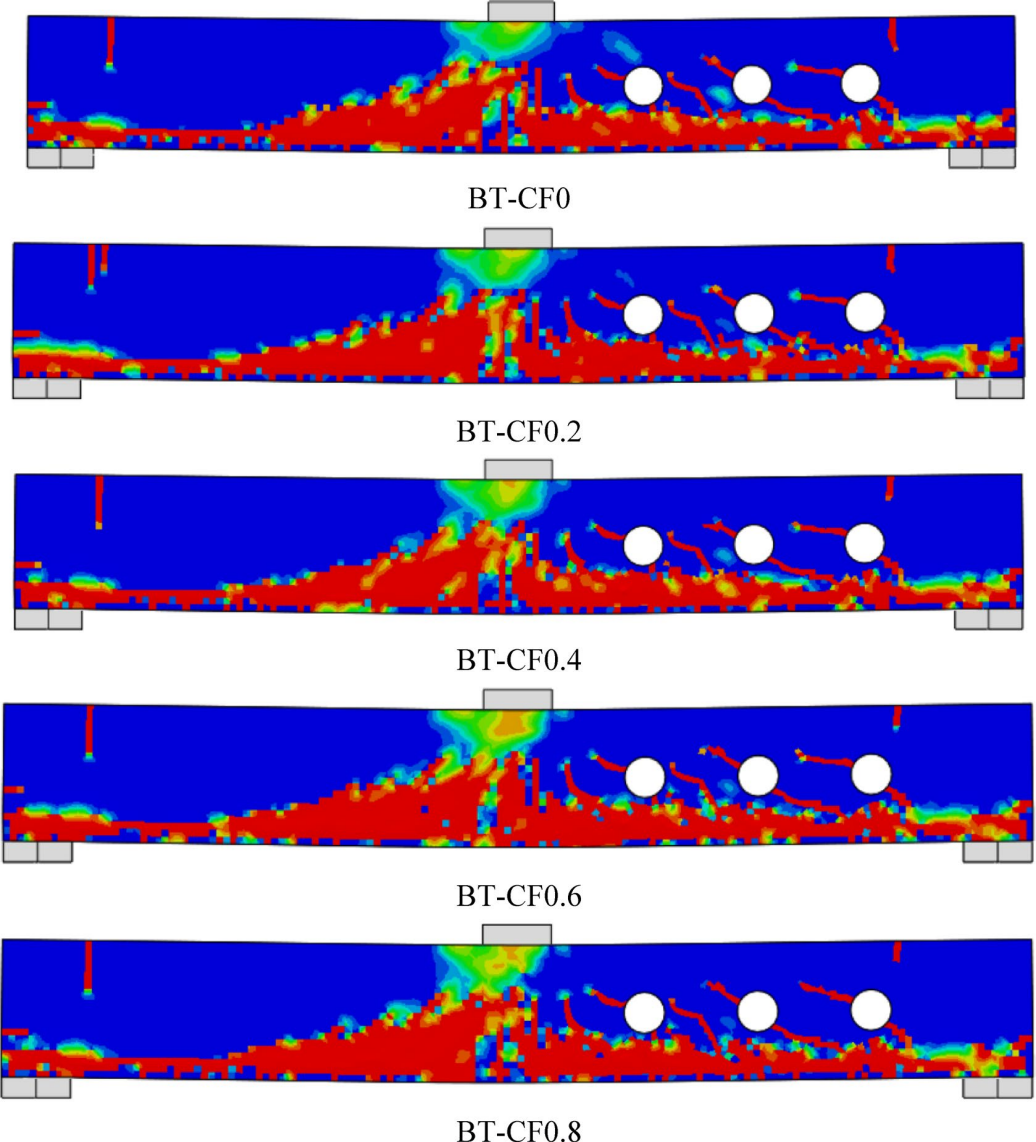
Failure patterns exhibited by Group GIV beams.

**Fig. 12:**
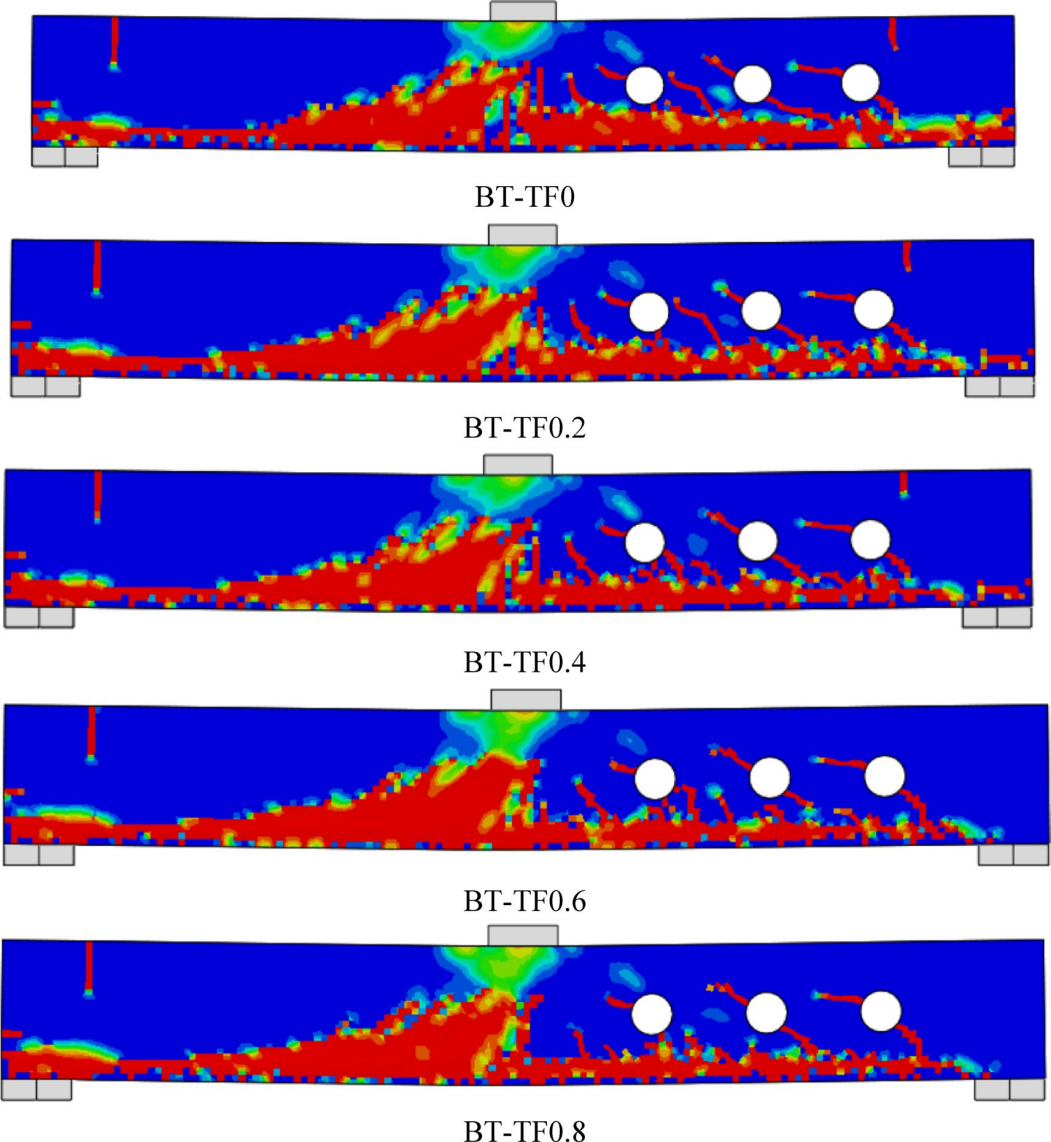
Failure patterns exhibited by Group GV beams.

**Fig. 13:**
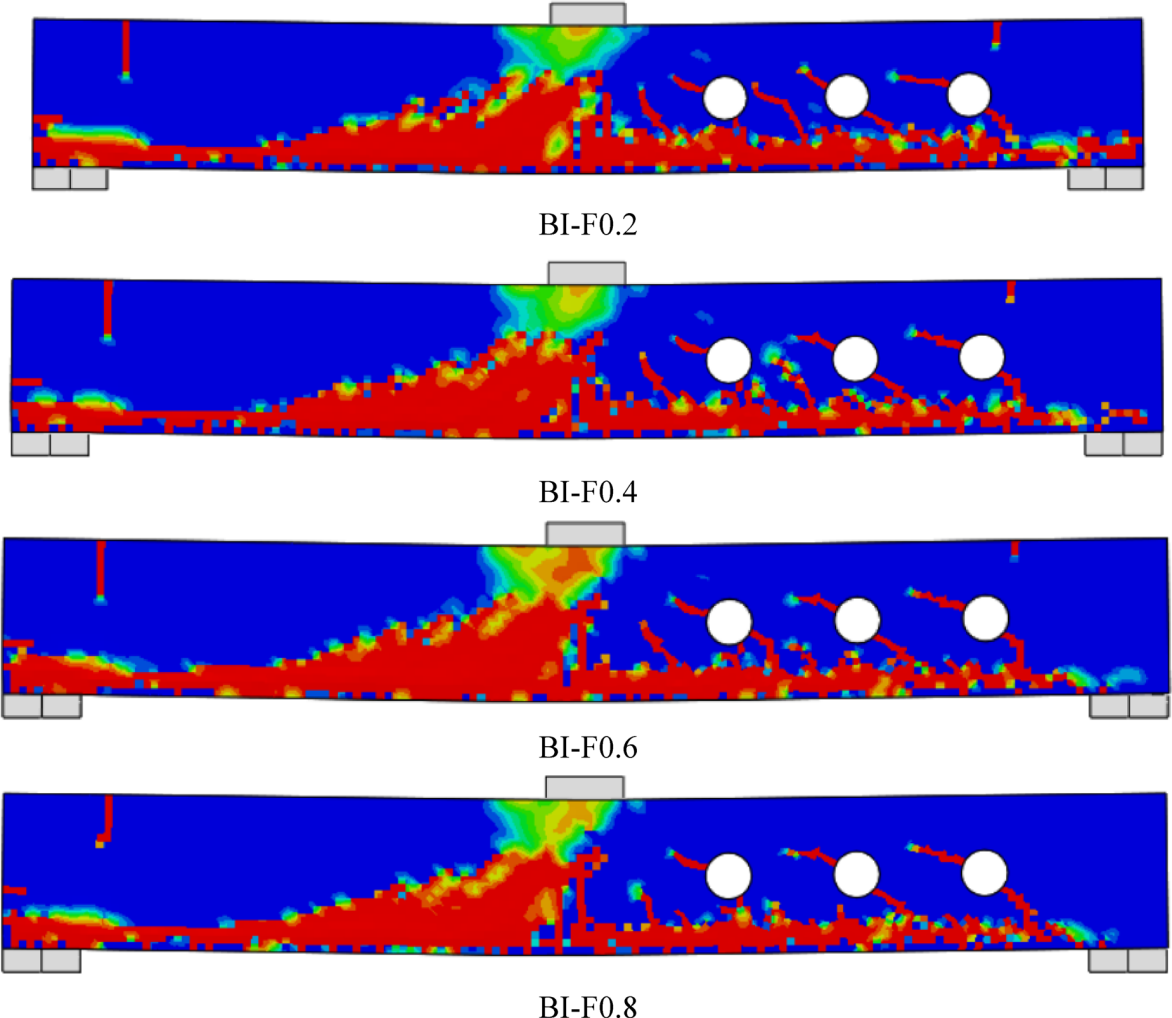
Failure patterns exhibited by Group GVI beams.

As shown in Figs. 10, 11, 12 and 13, all tested beams experienced shear failure, evidenced by distinct diagonal cracks extending from the load application points toward the supports. The experimental setup deliberately incorporated over-reinforcement in flexure to prevent bending-induced failure and isolate the influence of built-up steel reinforcement—namely, I-section and T-section configurations—on shear performance. The absence of any flexural cracks in the specimens confirms the effectiveness of this design strategy. In the control beam (B), which was constructed without web openings or shear reinforcement, the first diagonal shear crack emerged at a load of approximately 20 kN within the shear span. As the load increased, the beam exhibited a sudden and brittle shear failure. For the beams with multiple web openings, regardless of the reinforcement configuration (I-section or T-section), shear failure predominantly occurred on the right-hand side of the specimens. Diagonal cracking initiated at the corners of the openings—typically the most vulnerable stress concentration points—and progressed toward the load and support regions. As loading advanced, these cracks widened and additional shear cracks developed outside the perforation zone. These observations highlight the critical role of opening and reinforcement detailing in influencing shear crack propagation and overall failure behavior.

### Load–deflection curves

**Fig. 14:**
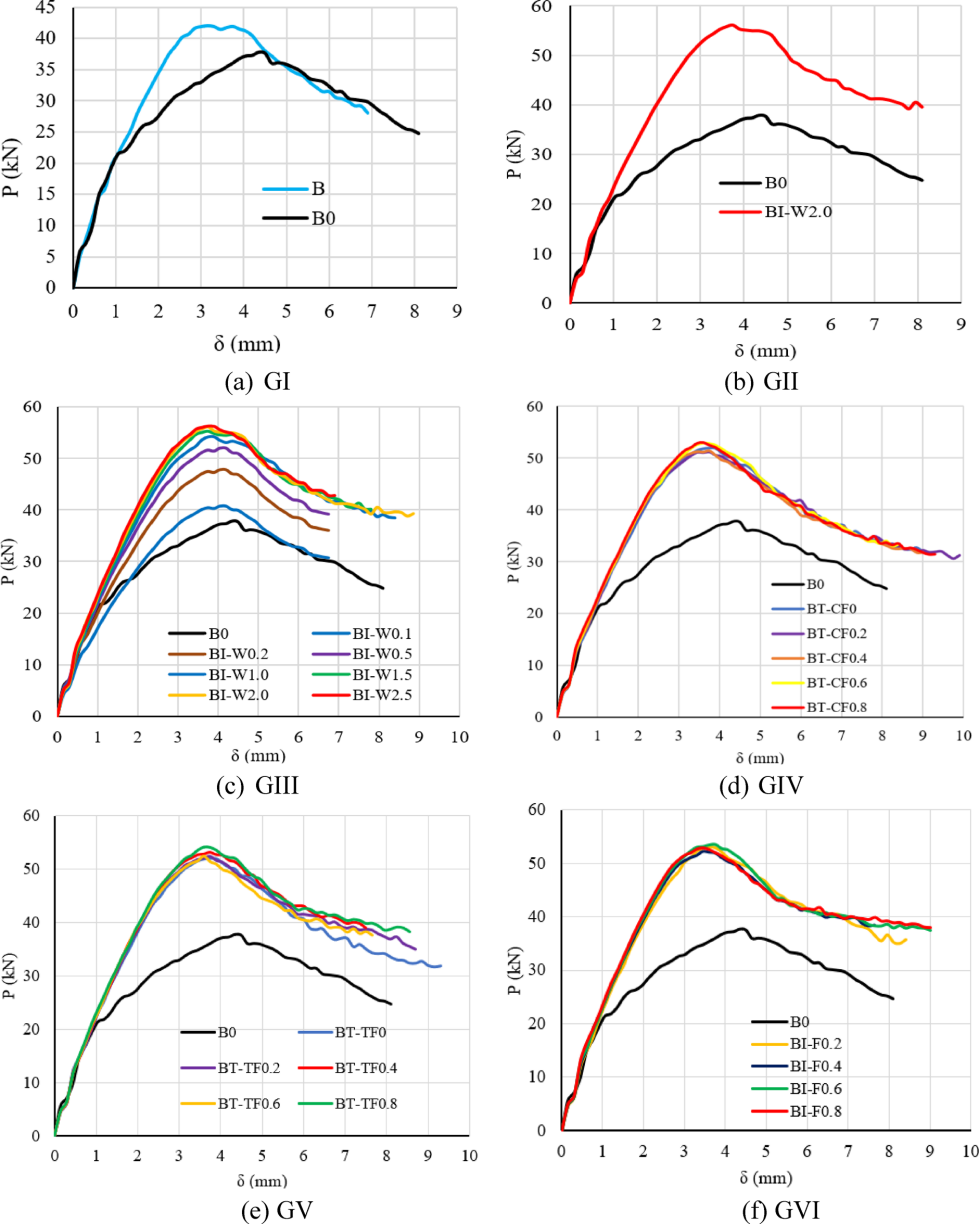
Load–deflection curves of all beams.

The load–deflection (P–δ) behavior of the tested reinforced concrete (RC) beams exhibited notable variations in stiffness, ductility, and ultimate strength, primarily influenced by the type and configuration of the integrated built-up steel sections, as presented in Fig. 14. Beams strengthened with I- and T-section steel elements generally demonstrated enhanced stiffness and greater load-carrying capacity in comparison to the unreinforced control specimens. The monotonic load–deflection curves consistently revealed three distinct behavioral phases. The first phase was linear and elastic, extending up to the initiation of the first visible cracking. This was followed by a nonlinear phase characterized by progressive crack propagation and cumulative damage, ultimately reaching the peak load. The differences observed during this stage underscore the significant role of reinforcement detailing. Beyond the peak, the post-ultimate phase was marked by either a plateau or a gradual reduction in load with continued deflection, reflecting structural softening and energy dissipation mechanisms.

The control beam with openings (B0) demonstrated a relatively lower deflection at failure (3.30 mm) compared to the solid control beam (B, 4.65 mm), despite carrying a higher load. This suggests a more brittle shear failure mode induced by stress concentration around the web openings. Beam B, lacking reinforcement and openings, exhibited more ductile behavior due to distributed cracking under flexural action.

Beams with I-section reinforcement, particularly BI-W2.0, showed a significant increase in load capacity with a moderate reduction in deflection, confirming the effectiveness of the I-shaped web in enhancing shear resistance without severely compromising ductility. As the web thickness increased from 0.1 to 2.5 mm, a consistent reduction in deflection was observed, indicating improved stiffness and crack control.

T-section reinforced beams displayed a similar trend, where increased flange width—both in compression and tension flanges—led to reduced deflections (as low as 3.70 mm) and enhanced load-carrying capacity. This reflects the increased moment of inertia and improved resistance to shear deformation. Overall, the introduction of built-up steel sections effectively enhanced both the strength and stiffness of the beams, with deflection behavior closely tied to geometric properties of the reinforcing elements.

### Overall analysis

The P–δ response of each beam was analyzed to extract key structural performance parameters. The ultimate load capacity (${P}_{u}$) was identified at the peak of the curve, with the corresponding deflection (${\delta }_{u}$) recorded at the same point. These parameters, grouped according to the numerical classifications, are presented in Table 4.

**Table 4:** Results of tested beams.

Group	Beam	${P}_{u}$(kN)	Gain in ${P}_{u}$ (%)	δu (mm)	Gain in δu (%)
GI; Effect of existing multi-openings	B	41.90	0.00	3.30	0.00
	B0	36.04	-13.99	4.65	40.91
GII; Effect existing of the I -Sec	B0	36.04	0.00	4.65	0.00
	BI-W2.0	55.30	53.44	3.90	-16.13
GIII; Effect of web thickness of the I -Sec	B0	36.04	0.00	4.65	0.00
	BI-W0.1	40.00	10.99	3.90	-16.13
	BI-W0.2	47.00	30.41	3.90	-16.13
	BI-W0.5	51.30	42.34	3.90	-16.13
	BI-W1.0	52.10	44.56	3.91	-15.91
	BI-W1.5	54.20	50.39	3.88	-16.56
	BI-W2.0	55.30	53.44	3.90	-16.13
	BI-W2.5	55.90	55.11	4.04	-13.12
GIV; Effect of compression flange width of the T -Sec	B0	36.04	0.00	4.65	0.00
	BT-CF0	52.40	45.39	3.75	-19.35
	BT-CF0.2	52.40	45.39	3.80	-18.28
	BT-CF0.4	52.60	45.95	3.81	-18.06
	BT-CF0.6	52.65	46.09	3.80	-18.28
	BT-CF0.8	52.76	46.39	3.90	-16.13
GV; Effect of tension flange width of the T -Sec	B0	36.04	0.00	4.65	0.00
	BT-TF0	51.40	42.62	3.70	-20.43
	BT-TF0.2	51.45	42.76	3.77	-18.92
	BT-TF0.4	52.30	45.12	3.80	-18.28
	BT-TF0.6	54.10	50.11	3.83	-17.63
	BT-TF0.8	55.10	52.89	3.90	-16.13
GVI; Effect of flange width of the I -Sec	B0	36.04	0.00	4.65	0.00
	BI-F0.2	52.50	45.67	3.60	-22.58
	BI-F0.4	52.30	45.12	3.66	-21.29
	BI-F0.6	52.10	44.56	3.70	-20.43
	BI-F0.8	52.10	44.56	3.70	-20.43

#### Effect of existing multi-openings (group GI)

Group GI serves as the baseline series, designed to evaluate the influence of multiple web openings on the structural performance of RC beams. The experimental results demonstrated a notable difference in both strength and deformation characteristics between the two specimens (Beam B and Beam B0). Beam B exhibited an ultimate load capacity (${P}_{u}$) of 41.90 kN and an ultimate deflection (δu) of 3.30 mm. In comparison, Beam B0 recorded a reduced ultimate load of 36.04 kN, representing a 13.99% decrease in strength, while its deflection increased to 4.65 mm, marking a 40.91% gain in ductility. These results clearly illustrate the detrimental impact of web openings on shear performance and load-carrying capacity. The significant reduction in load capacity observed in Beam B0 is primarily attributed to the disruption of the concrete web, which is critical in transferring shear forces. The presence of multiple openings introduces zones of stress concentration and weakens the continuity of the shear path, leading to premature cracking and early failure. This behavior underscores the inherent vulnerability of RC beams with unreinforced openings and the consequent reduction in structural efficiency.

Interestingly, Beam B0 also exhibited a considerable increase in ultimate deflection, suggesting enhanced ductility. The redistribution of stresses around the openings likely permitted larger deformations prior to failure, indicating a more ductile failure mode. While this characteristic may be favorable for energy dissipation, particularly in seismic applications, it also raises concerns regarding serviceability limits and long-term deflection under load.

Overall, the findings from Group GI highlight the necessity of introducing effective reinforcement strategies when incorporating service openings into RC beams. Although such openings are often unavoidable in practice, their impact on both strength and stiffness must be carefully addressed. The outcomes of this group establish a critical benchmark for evaluating the efficacy of built-up steel reinforcement elements, which are explored in subsequent groups. This comparison also reinforces the importance of understanding the trade-offs between strength reduction and ductility enhancement in the design of perforated RC members.

#### Effect existing of the I -Sec. (group GII)

Group GII was designed to evaluate the structural performance of reinforced concrete (RC) beams with multiple web openings, reinforced using built-up I-section (I-Sec) steel elements. This group builds upon the baseline established in Group GI by investigating the effectiveness of steel reinforcement in mitigating the adverse effects of these openings. The results reveal a significant improvement in the structural behavior of the reinforced beam. Beam B0, which contains unreinforced web openings, exhibited an ${P}_{u}$ of 36.04 kN and an δu of 4.65 mm. In contrast, the strengthened Beam BI-W2.0 achieved a substantially higher ultimate load of 55.30 kN—representing a 53.44% increase in load-carrying capacity**.** This impressive gain clearly demonstrates the effectiveness of I-section steel elements in restoring and even enhancing the shear performance of beams compromised by web openings. The substantial increase in strength is attributed to the presence of the I-section**,** which provides an alternative load path across the disrupted web area, effectively bridging the openings and restoring shear continuity. The steel web and flanges contribute to improved stiffness and crack control, delaying the onset of diagonal cracking and enhancing the overall capacity of the beam. The reinforcement not only offsets the strength loss due to openings but also results in a net performance gain over the unstrengthened configuration.

Interestingly, the ultimate deflection of BI-W2.0 was slightly reduced to 3.90 mm—a 16.13% decrease compared to B0. While this reduction indicates a decrease in ductility, it is consistent with the expected behavior of strengthened members, where the added stiffness from steel elements limits deformation. Though ductility is marginally reduced, the beam still exhibits acceptable deformability and avoids brittle failure, which is critical for structural safety.

In conclusion, Group GII confirms that the use of built-up I-section steel reinforcement is an effective strategy for compensating for the negative effects of service openings in RC beams. The reinforced specimen demonstrated not only a significant strength recovery but also a controlled and stable deformation response. These findings support the integration of such reinforcement techniques into design practices for beams with required web perforations, providing a practical solution for maintaining structural integrity while accommodating modern service demands.

#### Effect of web thickness of the I -Sec. (group GIII)

**Fig. 15:**
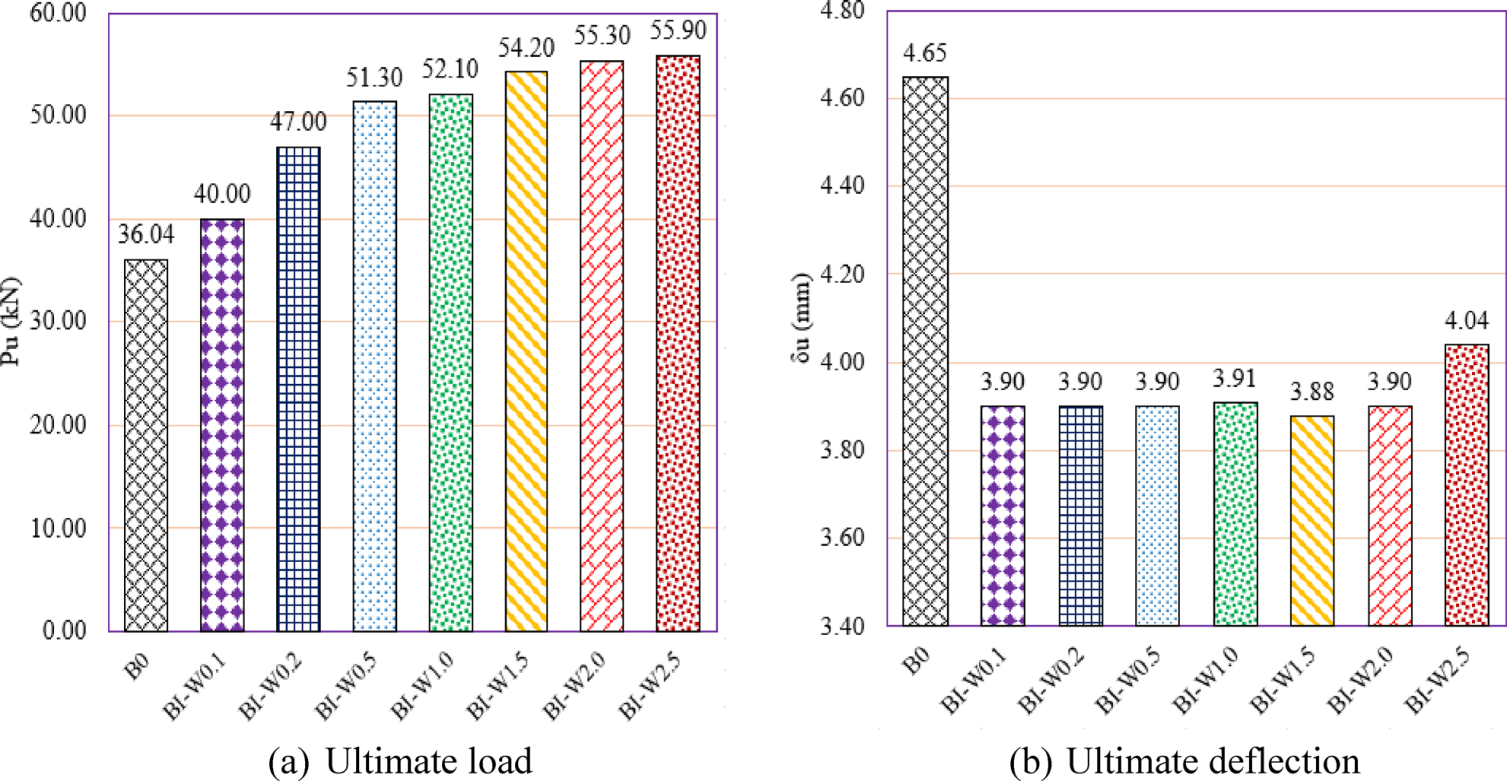
Effect of web thickness of the I -Section on the shear performance of tested beams.

Group GIII was designed to examine the influence of varying web thickness in built-up I-section steel reinforcement on the structural behavior of RC beams containing multiple web openings (Fig. 15). The goal was to identify the minimum effective web thickness that can restore or enhance the shear performance without unnecessary material use. The unstrengthened beam B0 exhibited an ${P}_{u}$ of 36.04 kN and a corresponding δu of 4.65 mm. With the introduction of I-section steel reinforcement, even the specimen with the thinnest web (BI-W0.1) showed an increase of 10.99% in load capacity, reaching 40.00 kN, though it exhibited a reduced deflection of 3.90 mm (a 16.13% decrease). As the web thickness increased, so did the structural performance. For instance, BI-W0.5 achieved a 42.34% gain in ${P}_{u}$, and BI-W1.5 recorded a 50.39% gain, indicating that increasing the steel web thickness significantly improves shear resistance by reinforcing the weakened web region adjacent to the openings. The peak performance was observed in BI-W2.5, which achieved the highest ultimate load of 55.90 kN, corresponding to a 55.11% increase over B0. Interestingly, the gains between BI-W2.0 (55.30 kN) and BI-W2.5 (55.90 kN) were marginal, suggesting a diminishing return in strength gain beyond a web thickness of 2.0 mm. This implies that 2.0 mm is likely an optimal web thickness**,** beyond which additional material contributes little to performance but increases cost and weight. In terms of ductility, all reinforced beams showed slightly reduced deflections compared to B0, with values clustered around 3.88–4.04 mm**,** representing an average decrease of about 15–16%**.** This is attributed to the increased stiffness introduced by the steel web, which limits deformation under loading. However, the deflection values remain within acceptable ranges, and none of the specimens’ exhibited signs of brittle failure, confirming that ductility was still preserved despite the stiffness increase.

In summary, Group GIII demonstrates that the web thickness of I-section reinforcement plays a critical role in restoring and enhancing the shear performance of RC beams with web openings. A minimum web thickness of 0.5–1.0 mm provides substantial strength recovery, while 2.0 mm appears to be the most efficient thickness for maximizing load capacity without excessive material use. These findings offer valuable insights for optimizing reinforcement strategies in perforated beams, promoting both structural efficiency and economic feasibility in modern RC design.

#### Effect of compression flange width of the T -Sec. (group GIV)

**Fig. 16:**
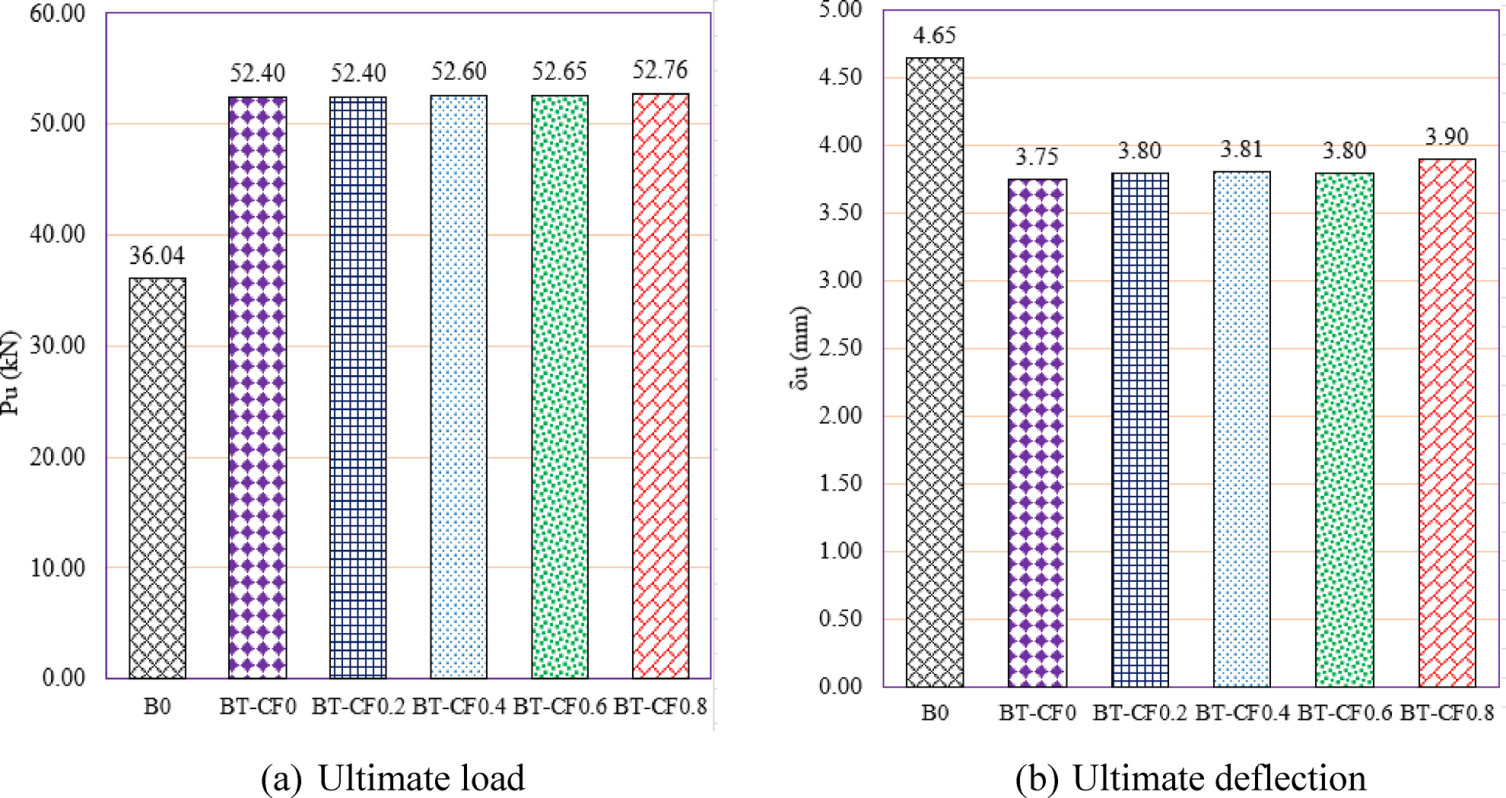
Effect of compression flange width of the T -Section on the shear performance of tested beams.

Group GIV focuses on evaluating the impact of compression flange width in T-section (T-Sec) steel reinforcement on the structural behavior of RC beams with multiple web openings (Fig. 16). The objective was to assess how increasing the compression flange width contributes to the enhancement of shear capacity and overall beam behavior, particularly through improved stress redistribution in the compression zone. The unreinforced reference beam, B0, recorded a ${P}_{u}$ of 36.04 kN and a δu of 4.65 mm. Upon reinforcing the beam with a T-section lacking a compression flange (BT-CF0), the ultimate load increased significantly to 52.40 kN, marking a 45.39% gain. This result indicates that even without a compression flange, the addition of the T-section reinforcement greatly improved shear performance by bridging the weakened web area and providing an alternative load path around the openings. However, this initial reinforcement also caused a 19.35% reduction in deflection, which reflects the increased stiffness of the strengthened section.

As the compression flange width was gradually increased—ranging from 16 mm (BT-CF0.2) to 64 mm (BT-CF0.8)—only marginal improvements in strength were observed. For example, BT-CF0.2 and BT-CF0.6 both maintained a ${P}_{u}$ of approximately 52.40–52.65 kN, while the maximum recorded strength was 52.76 kN in BT-CF0.8, representing a 46.39% increase over the unreinforced beam. This suggests that, while the presence of a compression flange has some positive influence on load capacity, the additional width beyond a certain point offers diminishing returns. The marginal differences (within ~ 1%) in ${P}_{u}$ across increasing flange widths indicate that most of the benefit is realized with the presence of any flange, and further increases primarily contribute to local stress distribution rather than global capacity enhancement.

In terms of ductility, all reinforced beams in this group exhibited a decrease in ultimate deflection compared to B0, with reductions ranging from 16.13% to 19.35%. The lowest deflection was seen in BT-CF0 (3.75 mm), while BT-CF0.8 recorded a slightly higher deflection of 3.90 mm, indicating a trend toward improved deformability as the flange width increased. The gradual recovery in deflection with increasing compression flange width may be attributed to improved distribution of compressive stresses, reducing localized crushing and allowing for slightly more deformation prior to failure.

In conclusion, Group GIV confirms that the inclusion of T-section steel reinforcement, even without a compression flange, significantly enhances the load-bearing capacity of RC beams with web openings. While the introduction of a compression flange provides marginal additional strength gains, it contributes to stiffness control and improved stress distribution. The findings suggest that a minimal flange width may be sufficient for practical design purposes, optimizing material use while maintaining performance. These results are highly relevant for structural engineers seeking efficient strengthening solutions in RC beams compromised by service openings.

#### Effect of tension flange width of the T -Sec. (group GV)

**Fig. 17:**
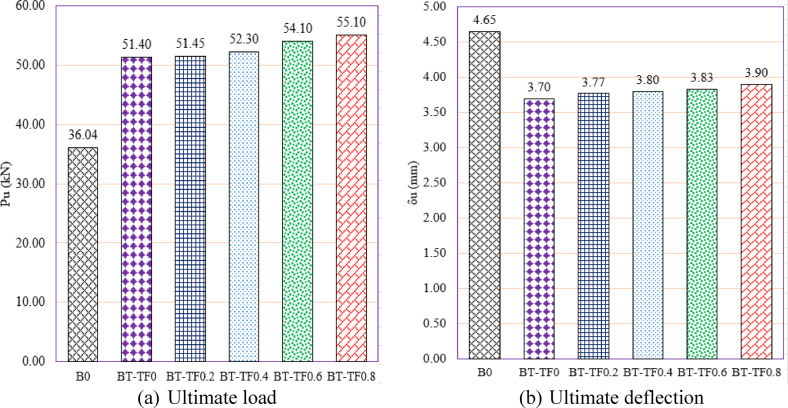
Effect of tension flange width of T -Section on the shear performance of tested beams.

Group GV investigates the effect of varying the tension flange width in T-section steel reinforcement on the structural performance of RC beams containing multiple web openings (Fig. 17). The primary aim was to determine how widening the bottom flange, located in the tensile region of the beam, influences load capacity and ductility. The unreinforced beam B0, used as a baseline, exhibited a ${P}_{u}$ of 36.04 kN and a corresponding δu of 4.65 mm. When reinforced with a T-section lacking a tension flange (BT-TF0), the load capacity increased markedly to 51.40 kN, representing a 42.62% improvement, while the deflection decreased to 3.70 mm (a 20.43% reduction). This demonstrates that even the vertical web component of the T-section alone provides substantial strength recovery by bridging shear-critical regions around the openings.

As the tension flange width was increased from 16 mm (BT-TF0.2) to 64 mm (BT-TF0.8), a consistent and notable improvement in ultimate load was observed. For instance, BT-TF0.4 reached 52.30 kN (a 45.12% gain), and BT-TF0.6 and BT-TF0.8 reached 54.10 kN and 55.10 kN, corresponding to 50.11% and 52.89% gains, respectively. These results highlight the effectiveness of a wider tension flange in resisting tensile forces, delaying crack propagation, and enhancing overall shear performance. The tension flange effectively engages in flexural and shear action, redistributing stresses and contributing to a more robust and resilient response.

Regarding ductility, all specimens in this group exhibited reduced ultimate deflections relative to the unreinforced beam, a trend consistent with the increase in stiffness provided by the T-section reinforcement. Deflection values ranged from 3.70 to 3.90 mm, reflecting reductions of approximately 16% to 20%. Notably, the deflection tended to recover slightly as the tension flange width increased. For example, BT-TF0.8 exhibited a deflection of 3.90 mm, suggesting that the wider tension flange allowed for improved deformation capacity while still enhancing strength, likely due to better stress distribution and delayed yielding in the tensile zone.

In summary, the results of Group GV underscore the importance of tension flange geometry in strengthening RC beams with web openings. Increasing the flange width led to progressive improvements in load capacity, with optimal gains observed at 64 mm width. While some ductility was sacrificed due to added stiffness, the deflection values remained within acceptable limits, maintaining a balance between strength and deformability. These findings provide valuable design guidance for selecting T-section reinforcement configurations that maximize structural efficiency, especially in beams where service openings compromise traditional load paths.

#### Effect of flange width of the I -Sec. (group GVI)

**Fig. 18:**
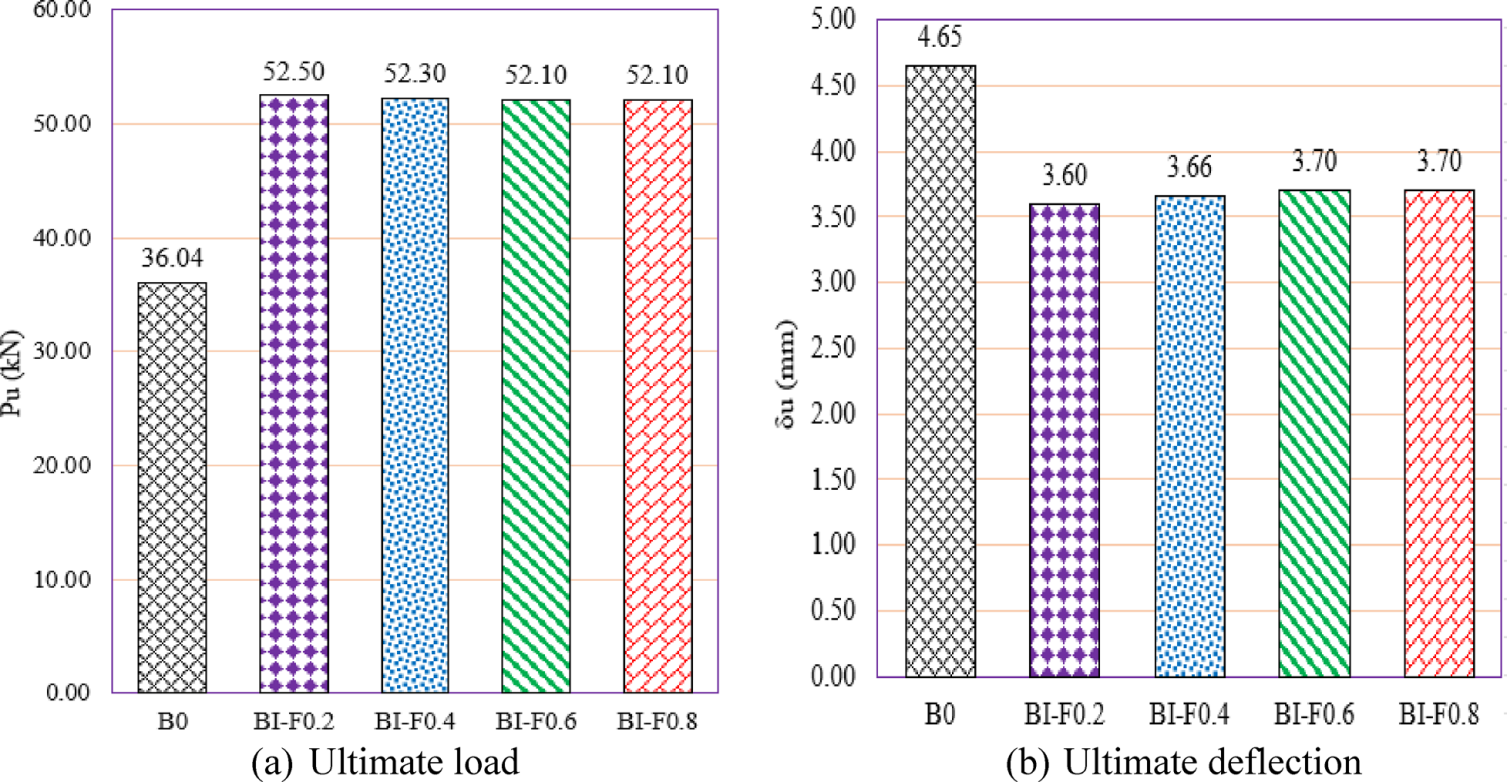
Effect of flange width of I -Section on the shear performance of tested beams.

Group GVI investigates the influence of flange width variation in I-section (I-Sec) steel reinforcement on the structural performance of RC beams with multiple web openings (Fig. 18). This group systematically varies the flange width from 16 to 64 mm while maintaining constant web dimensions (176 mm length × 2.0 mm thickness) to evaluate the contribution of flange geometry to load capacity and deformation behavior. The baseline unreinforced beam, B0, exhibited a ${P}_{u}$ of 36.04 kN and a δu of 4.65 mm. Upon strengthening with the I-section reinforcement featuring the narrowest flange width of 16 mm (BI-F0.2), the beam’s load capacity increased markedly to 52.50 kN, representing a 45.67% gain, accompanied by a reduction in deflection to 3.60 mm (a 22.58% decrease). This substantial enhancement underscores the effectiveness of the steel flange in restoring shear strength lost due to web openings by providing additional resistance against shear forces and improving load transfer. As the flange width increased to 32 mm (BI-F0.4), the ultimate load slightly decreased to 52.30 kN (a 45.12% gain), and deflection increased marginally to 3.66 mm, yet remained significantly below the baseline. For the larger flange widths of 48 mm (BI-F0.6) and 64 mm (BI-F0.8), the ultimate load stabilized around 52.10 kN (approximately 44.56% improvement), with corresponding deflections of 3.70 mm, indicating a consistent performance plateau regardless of further flange width increase.

The near-constant ultimate load values at flange widths beyond 32 mm suggest that increasing flange width beyond a moderate dimension provides minimal additional strength benefit. This plateau may be attributed to the effective engagement of the steel flange in shear resistance being maximized at intermediate widths, beyond which the structural response is controlled more by the web and flange thickness than by flange width alone. Meanwhile, the modest increase in deflection with wider flanges implies a slight reduction in stiffness but without compromising overall ductility significantly.

In summary, Group GVI demonstrates that the flange width of I-section reinforcement plays a vital role in enhancing shear capacity of RC beams with web openings. A flange width of approximately 16–32 mm suffices to achieve substantial gains in ultimate load and stiffness, with wider flanges offering limited further improvement. These findings are critical for optimizing the design of built-up I-section reinforcements, balancing material efficiency and structural performance for practical engineering applications.

## Conclusion

This study investigated the structural performance of reinforced concrete (RC) beams with multiple web openings, strengthened using built-up I-section and T-section steel elements as an alternative to conventional shear reinforcement. Through a combined approach of experimental testing and validated nonlinear finite element analysis (FEA), the research addresses a critical gap in the understanding of composite beams with service openings. The key findings are summarized as follows:

- The presence of multiple web openings significantly reduces the shear capacity of RC beams (by approximately 14% in this study) due to the disruption of the shear transfer path and high stress concentrations at the opening corners. Interestingly, while strength decreases, ductility (ultimate deflection) can increase by over 40%**,** indicating a shift towards a more brittle failure mode focused around the openings.
- The incorporation of built-up steel I-sections and T-sections proved to be a highly effective strategy for mitigating the adverse effects of web openings. The strengthened beam (BI-W2.0), which utilized a specific I-Sect. (176 × 2 mm web, 40 × 2 mm flanges), exhibited a remarkable 53.4% increase in ultimate load capacity compared to the unreinforced beam with openings (B0). This not only recovered the lost strength but also significantly enhanced the capacity beyond that of the solid beam.
- The web thickness of the I-section is a crucial parameter. Strength gains increased with web thickness up to 2.0 mm, beyond which further increases (e.g., to 2.5 mm) yielded only marginal returns, indicating a point of diminishing returns. A thickness of 2.0 mm was identified as optimal for balancing performance with material efficiency.
- Increasing the flange width of the I-section provided significant strength gains initially, but widths beyond 32 mm offered minimal additional benefit, resulting in a performance plateau.
- The width of the tension flange (located at the bottom of the beam) had a more pronounced and progressive impact on enhancing shear capacity (up to ~ 53% gain) compared to the compression flange. Increasing the compression flange width provided only marginal strength improvements.
- Even a T-section without flanges (just a vertical web) provided a substantial strength increase (~ 43%), highlighting the critical role of the web in bridging openings and restoring shear transfer.
- The addition of steel reinforcement invariably increased the stiffness of the beams, resulting in a reduction of ultimate deflection (by 16–22%) compared to the unreinforced beam with openings. This represents a trade-off, where significant gains in strength and stiffness are achieved with a controlled reduction in ductility, still maintaining acceptable deformation levels and avoiding brittle failure.
- The finite element model developed in ABAQUS demonstrated excellent accuracy in predicting the load–deflection response and failure modes of the tested beams, with an average experimental-to-simulation ratio of 0.953 for peak load and 0.967 for deflection. This validated model provided a reliable tool for the extensive parametric study.
- It is recommended to investigate beams with steel sections (I- or T-sections) embedded along openings on one or both sides. This would provide further insight into the optimal reinforcement placement and effectiveness for varying opening configurations.

This research demonstrates that built-up I-section and T-section steel elements are a highly effective and viable solution for strengthening RC beams with web openings. The findings provide practical, optimized design guidelines for structural engineers, enabling the safe and efficient integration of essential service systems in modern buildings without compromising structural safety and performance. The study effectively bridges the gap between architectural functionality and structural integrity.

## Data Availability

The raw data required to reproduce these findings are available only with direct contact through email to the corresponding author. The processed data required to reproduce these findings are available only with direct contact through email to the corresponding author.
